# Repair of Acute Respiratory Distress Syndrome by Stromal Cell Administration in COVID-19 (REALIST-COVID-19): A structured summary of a study protocol for a randomised, controlled trial

**DOI:** 10.1186/s13063-020-04416-w

**Published:** 2020-06-03

**Authors:** Ellen Gorman, Manu Shankar-Hari, Phil Hopkins, William S. Tunnicliffe, Gavin D. Perkins, Jonathan Silversides, Peter McGuigan, Colette Jackson, Roisin Boyle, Jamie McFerran, Cliona McDowell, Christina Campbell, Margaret McFarland, Jon Smythe, Jacqui Thompson, Barry Williams, Gerard Curley, John G. Laffey, Mike Clarke, Cecilia O’Kane, Daniel F. McAuley

**Affiliations:** 1 Wellcome Wolfson Institute for Experimental Medicine, School of Medicine, Dentistry and Biomedical Science Queen’s University Belfast, Belfast, UK; 2 Guy’s and St Thomas’ NHS Foundation Trust London and School of Immunology and Microbial Sciences, King’s College London, London, UK; 3 Research and Development lead in Critical Care, Kings Trauma Centre, King’s College London, London, UK; 4 Queen Elizabeth Hospital, Birmingham, UK; 5 University of Warwick, Coventry, UK; 6 Belfast Health and Social Care Trust, Belfast, UK; 7 Royal Victoria Hospital, Belfast, UK; 8 Northern Ireland Clinical Trials Unit, Belfast, UK; 9 NHS Blood and Transplant, Birmingham, UK; 10 Independent Public and Patient Representative, Sherborne, UK; 11 Royal College of Surgeons in Ireland, Dublin, Ireland; 12 National University of Ireland, Galway, Ireland; 13 School of Medicine, Dentistry and Biomedical Sciences, Queen’s University Belfast, Belfast, UK

**Keywords:** COVID-19, Randomised controlled trial, protocol, Mesenchymal Stromal Cells, Mesenchymal Stem Cells, MSCs, Acute Respiratory Distress Syndrome, ARDS

## Abstract

**Objectives:**

The primary objective of the study is to assess the safety of a single intravenous infusion of Mesenchymal Stromal Cells (MSCs) in patients with Acute Respiratory Distress Syndrome (ARDS) due to COVID-19. Secondary objectives are to determine the effects of MSCs on important clinical outcomes, as described below.

**Trial design:**

REALIST COVID 19 is a randomised, placebo-controlled, triple blinded trial.

**Participants:**

The study will be conducted in Intensive Care Units in hospitals across the United Kingdom. Patients with moderate to severe ARDS as defined by the Berlin definition, receiving invasive mechanical ventilation and with a diagnosis of COVID-19 based on clinical diagnosis or PCR test will be eligible.

Patients will be excluded for the following reasons: more than 72 hours from the onset of ARDS; age < 16 years; patient known to be pregnant; major trauma in previous 5 days; presence of any active malignancy (other than non-melanoma skin cancer); WHO Class III or IV pulmonary hypertension; venous thromboembolism currently receiving anti-coagulation or within the past 3 months; patient receiving extracorporeal life support; severe chronic liver disease (Child-Pugh > 12); Do Not Attempt Resuscitation order in place; treatment withdrawal imminent within 24 hours; prisoners; declined consent; non-English speaking patients or those who do not adequately understand verbal or written information unless an interpreter is available; previously enrolled in the REALIST trial.

**Intervention and comparator:**

Intervention: Allogeneic donor CD362 enriched human umbilical cord derived mesenchymal stromal cells (REALIST ORBCEL-C) supplied as sterile, single-use cryopreserved cell suspension of a fixed dose of 400 x10^6^ cells in 40ml volume, to be diluted in Plasma-Lyte 148 to a total volume of 200mls for administration.

Comparator (placebo): Plasma-Lyte 148 Solution for Infusion (200mls).

The cellular product (REALIST ORBCEL-C) was developed and patented by Orbsen Therapeutics.

**Main outcomes:**

The primary safety outcome is the incidence of serious adverse events. The primary efficacy outcome is Oxygenation Index (OI) at day 7. Secondary outcomes include: OI at days 4 and 14; respiratory compliance, driving pressure and PaO_2_/FiO_2_ ratio (PF ratio) at days 4, 7 and 14; Sequential Organ Failure Assessment (SOFA) score at days 4, 7 and 14; extubation and reintubation; ventilation free days at day 28; duration of mechanical ventilation; length of ICU and hospital stay; 28-day and 90-day mortality.

**Randomisation:**

After obtaining informed consent, patients will be randomised via a centralised automated 24-hour telephone or web-based randomisation system (CHaRT, Centre for Healthcare Randomised Trials, University of Aberdeen). Randomisation will be stratified by recruitment centre and by vasopressor use and patients will be allocated to REALIST ORBCEL-C or placebo control in a 1:1 ratio.

**Blinding (masking):**

The investigator, treating physician, other members of the site research team and participants will be blinded. The cell therapy facility and clinical trials pharmacist will be unblinded to facilitate intervention and placebo preparation. The unblinded individuals will keep the treatment information confidential. The infusion bag will be masked at the time of preparation and will be administered via a masked infusion set.

**Numbers to be randomised (sample size):**

A sample size of 60 patients with 30 patients randomised to the intervention and 30 to the control group. If possible, recruitment will continue beyond 60 patients to provide more accurate and definitive trial results. The total number of patients recruited will depend on the pandemic and be guided by the data monitoring and ethics committee (DMEC).

**Trial status:**

REALIST Phase 1 completed in January 2020 prior to the COVID-19 pandemic. This was an open label dose escalation study of REALIST ORBCEL-C in patients with ARDS. The COVID-19 pandemic emerged as REALIST Phase 2 was planned to commence and the investigator team decided to repurpose the Phase 2 trial as a COVID-19 specific trial. This decision was discussed and approved by the Trial Steering Committee (TSC) and DMEC. Submissions were made to the Research Ethics Committee (REC) and MHRA to amend the protocol to a COVID-19 specific patient population and the protocol amendment was accepted by the REC on 27^th^ March 2020 and MHRA on 30^th^ March 2020 respectively. Other protocol changes in this amendment included an increase in the time of onset of ARDS from 48 to 72 hours, inclusion of clinical outcomes as secondary outcomes, the provision of an option for telephone consent, an indicative sample size and provision to continue recruitment beyond this indicative sample size. The current protocol in use is version 4.0 23.03.2020 (Additional file 1). Urgent Public Health status was awarded by the NIHR on 2 April 2020 and the trial opened to recruitment and recruited the first participant the same day. At the time of publication the trial was open to recruitment at 5 sites across the UK (Belfast Health and Social Care Trust, King’s College London, Guys and St Thomas’ Hospital London, Birmingham Heartlands Hospital and the Queen Elizabeth Hospital Birmingham) and 12 patients have been recruited across these sites. Additional sites are planned to open and appropriate approvals for these are being obtained. It is estimated recruitment will continue for 6 months.

**Trial registration:**

ClinicalTrials.gov NCT 03042143 (Registered 3 Feb 2017).

EudraCT 2017-000585-33 (Registered 28 Nov 2017).

**Full protocol:**

The full protocol (version 4.0 23.03.2020) is attached as an additional file, accessible from the Trials website (Additional file 1). In the interest of expediting dissemination of this material, the familiar formatting has been eliminated; this Letter serves as a summary of the key elements of the full protocol.

The study protocol has been reported in accordance with the Standard Protocol Items: Recommendations for Clinical Interventional Trials (SPIRIT) guidelines (Additional file 2).

## Supplementary information

**Additional file 1.** The REALIST Study Repair of Acute Respiratory Distress Syndrome by Stromal Cell Administration Protocol v 4.0.

**Additional file 2.** SPIRIT 2013 Checklist: Recommended items to address in a clinical trial protocol and related documents*.

## Data Availability

The investigator team will have full access to the final trial dataset. The trial will be reported in accordance with the Consolidated Standards of Reporting Trials (CONSORT) guidelines. The results of the trial will be published when data on the primary outcome are available. Long term data and mechanistic data will also be reported though may form the basis of separate publications. Publications will be published in peer-reviewed open access journals (in keeping with the Wellcome Trust open access policy). A lay person’s summary of the principal findings of the results will be sent to all patients involved in the study at their request.

