# Supplementary material for: Repair of Acute Respiratory Distress Syndrome by Stromal Cell Administration in COVID-19 (REALIST-COVID-19): A structured summary of a study protocol for a randomised, controlled trial
Source: Trials. 2020 Jun 3;21:462. doi: 10.1186/s13063-020-04416-w (PMC7267756; doi:10.1186/s13063-020-04416-w)
Supplement: Supplementary file 1 — Additional file 1. The REALIST Study Repair of Acute Respiratory Distress Syndrome by Stromal Cell Administration Protocol v 4.0. [file 13063_2020_4416_MOESM1_ESM.pdf]

The REALIST Study

Repair of Acute Respiratory Distress Syndrome by Stromal Cell Administration

Protocol Number:	16154DMcA-AS
Protocol Version:<br>*(See Summary of Key Changes Form for Differences From Last Version)*	4.0 Final
Protocol date	23.03.2020
Protocol amendment number	N/A
EudraCT Number:	2017-000584-33
ClinicalTrials.gov	NCT03042143
Ethics Reference Number:	18/NE/0006
Sources of monetary or material support	
Funder:	Wellcome Trust<br>Gibbs Building<br>215 Euston Road<br>London<br>NW1 2BE<br>United Kingdom<br><br>Research and Development Division of the Public Health Agency<br>Northern Ireland<br>12-22 Linenhall Street<br>Belfast<br>BT2 8BS<br>Northern Ireland
Sponsor Details	
Sponsor:	Belfast Health and Social Care Trust<br>The Royal Hospitals<br>Grosvenor Road<br>Belfast<br>BT12 6BA<br>N. Ireland
Chief Investigator:	Professor Danny McAuley<br>Queen’s University Belfast<br>Centre for Experimental Medicine<br>Welcome Wolfson Institute for Experimental Medicine<br>97 Lisburn Road<br>Belfast, BT9 7AE<br>N. Ireland

PROTOCOL AUTHORISATION

Protocol Title	Repair of Acute Respiratory Distress Syndrome by Stromal Cell Administration (REALIST)
Protocol Acronym (if applicable)	REALIST
Protocol Number	16154DMcA-AS
Protocol Version Number/Date	4.0 23.03.2020
Protocol Amendments	REALIST Protocol Final v3.0 26.06.2019 amended to REALIST Protocol Final v4.0_23.03.2020_Final

A review of the protocol has been completed and is understood and approved by the following:

Danny McAuley ______________________ ___ / ___ / ___

Chief Investigator Signature Date

Christina Campbell ______________________ ___ / ___ / ___

Statistician Signature Date

Table of Contents

The REALIST Study 1

PROTOCOL AUTHORISATION 2

Table of Contents 3

LIST OF ABBREVIATIONS 6

1 STUDY SUMMARY 8

2 STUDY TEAM 10

3 ROLES AND RESPONSIBILITIES 11

3.1 Funder 11
3.2 Sponsor 11
3.3 Trial Oversight Committees 11

4 BACKGROUND AND RATIONALE 13

4.1 Acute Respiratory Distress Syndrome 13
4.2 ARDS in COVID 19 13
4.3 Mesenchymal Stromal Cells 13
4.4 Pre-clinical evidence for MSC efficacy and safety in ARDS 14
4.5 Clinical evidence for MSC safety and efficacy in ARDS 14
4.6 Dose regimen rationale 15
4.7 Immunologic monitoring 15
4.8 Rationale for a trial of REALIST ORBCEL-C in ARDS 16

5 STUDY AIM AND OBJECTIVES 16

5.1 Research Hypothesis 16
5.2 Study Aim 16
5.3 Study Objectives 17

6 STUDY DESIGN 17

6.1 Study Design 17
6.2 Study Schematic Diagrams 18
6.3 Study Timeline 19
6.4 End of Study 20

7 STUDY OUTCOME MEASURES 21

7.1 Primary Outcome Measure 21
7.2 Secondary Outcome Measures 21

8 PATIENT ELIGIBILITY ... 21
8.1 Study Setting ... 21
8.2 Study Population ... 22
8.3 Eligibility Criteria ... 22
8.4 Co-enrolment guidelines ... 23
9 PATIENT SCREENING, CONSENT and RECRUITMENT ... 23
9.1 Patient Screening ... 23
9.2 Informed Consent Procedure ... 23
10 ASSIGNMENT OF INTERVENTION ... 25
10.1 Allocation to the Phase 1 Trial ... 25
10.2 Randomisation for the Phase 2 Trial ... 25
10.3 Blinding ... 25
10.4 Unblinding Procedure ... 26
11 INVESTIGATIONAL MEDICINAL PRODUCT ... 27
11.1 Study Intervention ... 27
11.2 Study Drug Supply ... 29
11.3 Study Drug Accountability ... 29
11.4 Study Drug Infusion ... 29
11.5 Study Drug Termination Criteria ... 29
11.6 Study Drug Return and Destruction ... 30
11.7 Concomitant Care ... 30
11.8 Management During the Study Drug Infusion ... 30
12 SCHEDULE OF ASSESSMENTS ... 31
12.1 Schedule of Assessments ... 31
12.2 Study Visits and Procedures ... 31
12.3 Sampling Procedures for Exploratory Outcomes ... 34
12.4 Follow Up Visits and Procedures ... 36
13 DATA COLLECTION AND MANAGEMENT ... 36
13.1 Data Collection ... 36
13.2 Data Management ... 36
13.3 Data Quality ... 37
14 STATISTICAL CONSIDERATIONS ... 37
14.1 Sample Size ... 37
14.2 Analysis Population ... 38
14.3 Statistical Methods ... 38
14.4 Missing Data ... 38

15 PHARMACOVIGILANCE 39
15.1 Definition of Adverse Events 39
15.2 Assessment of Adverse Events 39
15.3 Assessment of Causality 40
15.4 Assessment of Severity 41
15.5 Assessment of Seriousness 41
15.6 Assessment of Expectedness 41
15.7 Adverse Event Reporting Period 41
15.8 Adverse Event Reporting 42
15.9 Recording and Reporting Urgent Safety Measures 43
15.10 Pregnancy Reporting 43
16 DATA MONITORING 44
16.1 Data Access 44
16.2 Monitoring Arrangement 44
17 REGULATIONS, ETHICS AND GOVERNANCE 44
17.1 Regulatory and Ethical Approvals 44
17.2 Ethical Considerations 44
17.3 Protocol Compliance 45
17.4 Protocol Amendments 45
17.5 Good Clinical Practice 45
17.6 Indemnity 45
17.7 Patient Confidentiality 45
17.8 Data Access 46
17.9 Record Retention 46
17.10 Competing Interests 46
18 DISSEMINATION/PUBLICATIONS 46
18.1 Publication Policy 46
18.2 Authorship Policy 47
18.3 Data Sharing Statement 47
19 REFERENCES 48
20 APPENDICES 52
Appendix 1: P/F Ratio Reference Table for Inclusion Criteria 53
Appendix 2: ARDSNet PEEP/FiO2 Table 54
Appendix 3: Guidelines for Management During Study Drug Infusion 55

LIST OF ABBREVIATIONS

Abbreviation / Acronym	Full Wording
ABG	Arterial Blood Gas
AE	Adverse Event
ALI	Acute Lung Injury
APACHE	Acute Physiology and Chronic Health Evaluation
AR	Adverse Reaction
ARDS	Acute Respiratory Distress Syndrome
ATMP	Advanced Therapeutic Medicinal Product
BAL	Bronchoalveolar Lavage
BHSCT	Belfast Health and Social Care Trust
BM	Bone Marrow
CI	Chief Investigator
CFU-F	Colony Forming Unit Fibroblasts
CMP	Case Mix Programme
CO_2	Carbon Dioxide
CONSORT	Controlled Standards of Reporting Trials
COVID-19	Novel coronavirus (2019)
CPAP	Continuous positive airway pressure
CRF	Case Report Form
Crs	Respiratory compliance
CRP	C-reactive protein
CTA	Clinical Trial Authorisation
CTIMP	Clinical Trial Investigational Medicinal Product
CTU	Clinical Trials Unit
CXR	Chest X-ray
DMEC	Data Monitoring and Ethics Committee
DLT	Dose Limiting Toxicity
DMP	Data Management Plan
DMSO	Dimethyl Sulfoxide
DNAR	Do Not Attempt Resuscitation
ECLS	Extracorporeal Life Support
ECMO	Extracorporeal Membrane Oxygenation
EKG	Electrocardiogram
ELISA	Enzyme-Linked Immunosorbent Assay
EudraCT	European Clinical Trials Database
FiO_2	Fraction of Inspired Oxygen
GP	General Practitioner
GCP	Good Clinical Practice
GMP	Good Manufacturing Practice
HLA Ab	Human Leukocyte Antigen Anti-bodies
HTA	Human Tissue Authority
IB	Investigator Brochure
IMPD	Investigational Medicinal Product Dossier
ICH	International Conference on Harmonisation
ICNARC	Intensive Care National Audit & Research Centre
ICU	Intensive Care Unit
ISF	Investigator Site File
IV	Intravenous
LPS	Lipopolysaccharide
MDM	Monocyte-derived Macrophages

MHRA	Medicines and Healthcare products Regulatory Agency
MMP	Matrix metalloproteinases
MSC	Mesenchymal Stromal Cell
MTD	Maximal Tolerated Dose
NHS	National Health Service
NHSBT	National Health Service Blood and Transplant
NICTU	Northern Ireland Clinical Trials Unit
NETs	Neuroendocrine Tumors
NMBD	Neuromuscular Blocking Drugs
O_2	Oxygen
OI	Oxygenation Index
$PaCO_2$	Partial Pressure of Carbon Dioxide in arterial blood
PaO_2	Partial Pressure of Oxygen in arterial blood
PBW	Predicted Body Weight
PCR	Polymerase Chain Reaction
PerLR	Personal Legal Representative
PEEP	Positive End Expiratory Pressure
P/F ratio	PaO_2/FiO_2 ratio
PI	Principal Investigator
PIS	Patient Information Sheet
ProfLR	Professional Legal Representative
QUB	Queens University Belfast
RAGE	Receptor for Advanced Glycation Endproducts
REC	Research Ethics Committee
RR	Respiratory Rate
SAE	Serious Adverse Event
SAP	Statistical Analysis Plan
SAPS	Simplified Acute Physiology Score
SDV	Source Data Verification
SOFA	Sequential Organ Failure Assessment
SOPs	Standard Operating Procedures
SP-D	Surfactant Protein-D
SUSAR	Suspected Unexpected Serious Adverse Reaction
TMF	Trial Master File
TMG	Trial Management Group
TSC	Trial Steering Committee
UAR	Unexpected Adverse Reaction
VFD	Ventilator Free Days
WHO	World Health Organisation

1 STUDY SUMMARY

Scientific title	Repair of Acute Respiratory Distress Syndrome by Stromal Cell Administration (REALIST).
Public title	A trial of Mesenchymal Stromal Cells (MSCs) for acute respiratory failure
Health condition(s) or problem(s) studied	Acute Respiratory Distress Syndrome
Study Design	An open label dose escalation phase 1 trial followed by a randomised, double-blind, allocation concealed, placebo-controlled trial.
Study Aim and Objectives	The primary objective is to assess the safety of a single intravenous infusion of MSCs in patients with ARDS due to COVID-19.<br><br>Secondary objectives are to determine the effects of MSCs on:<br><br>1. Physiological indices of respiratory dysfunction reflecting severity of ARDS, as measured by oxygenation index (OI), respiratory compliance, and P/F ratio.<br>2. Sequential organ failure assessment (SOFA) score.<br>3. Extubation and reintubation.<br>4. Ventilation free days at day 28.<br>5. Duration of ventilation.<br>6. Length of ICU and hospital stay.<br>7. 28-day and 90-day mortality.
Study Intervention	The investigational cellular product will be allogeneic unrelated donor human umbilical cord-derived CD362 enriched MSCs (market name REALIST ORBCEL-C).<br><br>The dose escalation groups in the open label phase 1 trial will receive a single infusion of 100, 200 or 400 $x10^6$ cells.<br><br>In the phase 2 study, patients will receive 400 x 10^6 REALIST ORBCEL-C or Plasma-Lyte 148 (placebo).
Primary Safety Outcome	Safety of MSCs, as defined by the incidence of serious adverse events (SAEs).
Primary Efficacy Outcome	Oxygenation Index (OI) at day 7.
Secondary Outcomes	Oxygenation Index (OI) at days 4 and 14<br>Respiratory compliance and P/F ratio at days 4, 7 and 14<br>Sequential Organ Failure Assessment (SOFA) score at days 4, 7 and 14<br>Extubation and reintubation<br>Ventilation free days at day 28<br>Duration of ventilation<br>Length of ICU and hospital stay

	28-day and 90-day mortality
Inclusion and Exclusion Criteria	Inclusion<br>1. Moderate to severe ARDS as defined by the Berlin definition.<br>2. Patient is receiving invasive mechanical ventilation.<br>3. COVID-19 based on clinical diagnosis or PCR test.<br><br>Exclusion<br>1. More than 72 hours from the onset of ARDS.<br>2. Age < 16 years.<br>3. Patient is known to be pregnant.<br>4. Major trauma in the prior 5 days.<br>5. Presence of any active malignancy (other than non-melanoma skin cancer) that required treatment within the last year.<br>6. WHO Class III or IV pulmonary hypertension.<br>7. Venous thromboembolism currently receiving anti-coagulation or within the past 3 months.<br>8. Currently receiving extracorporeal life support (ECLS).<br>9. Severe chronic liver disease with Child-Pugh score > 12.<br>10. DNAR (Do Not Attempt Resuscitation) order in place.<br>11. Treatment withdrawal imminent within 24 hours.<br>12. Consent declined.<br>13. Prisoners.<br>14. Non-English speaking patients or those who do not adequately understand verbal or written information unless an interpreter is available.<br>15. Previously enrolled in the REALIST trial.
Study Setting	Adult intensive care units
Target Sample Size	Up to 18 participants for the phase 1 trial.<br><br>Indicative sample size of 60 participants for the phase 2 clinical trial (30 in intervention arm), but will continue to recruit beyond this if there is no clear problem with safety, no clear results on clinical outcomes and the necessary resources are available.
Study Duration	60 months (which includes 24 month follow up)

2 STUDY TEAM

Chief Investigator	**Professor Danny McAuley**<br>Queens University Belfast
Co-Investigators	**Dr Cecilia O'Kane**<br>Queens University Belfast
	**Professor John Laffey**<br>National University of Ireland, Galway
	**Professor Ger Curley**<br>Royal College of Surgeons in Ireland
	**Dr Jon Smythe**<br>NHS Blood and Transplant (NHSBT), Cellular Molecular Therapies, Oxford
	**Professor Michael Matthay**<br>University of California, San Francisco
Statistician	**Christina Campbell**<br>Northern Ireland Clinical Trials Unit (NICTU)
Clinical Trials Unit	**Northern Ireland Clinical Trials Unit (NICTU)**<br>1st Floor Elliott Dynes Building, The Royal Hospitals, Grosvenor Road, Belfast, BT12 6BA, N. Ireland
Sponsor	**Belfast Health and Social Care Trust**<br>**Alison Murphy**<br>Research Manager, Research Office, 2nd Floor King Edward Building, The Royal Hospitals, Grosvenor Road, Belfast, BT12 6BA, N. Ireland
Sponsor's Reference	16154DMcA-AS
Contact for Queries	**Clinical Trial Manager**<br>**Northern Ireland Clinical Trials Unit (NICTU)**<br>1st Floor Elliott Dynes Building, The Royal Hospitals,<br>Grosvenor Road,<br>Belfast, BT12 6BA<br>N. Ireland<br>Email: REALIST@nictu.hscni.net

3 ROLES AND RESPONSIBILITIES

3.1 Funder

The Wellcome Trust Health Innovation Challenge Fund and the Research and Development Division of the Public Health Agency, Northern Ireland will provide the research costs for the REALIST study. The funders have no role in the study design, data acquisition, data analysis, or manuscript preparation.

3.2 Sponsor

The Belfast Health and Social Care Trust (BHSCT) will act as Sponsor for the study and the Chief Investigator (CI) will take overall responsibility for the conduct of the trial. Separate agreements will be put in place between the Sponsor and each organisation undertaking Sponsor delegated duties in relation to the management of the study.

3.3 Trial Oversight Committees

Trial Management Group (TMG)

A Trial Management Group (TMG) will be established and Chaired by the CI. It will have representatives from the Clinical Trials Unit (CTU) and other co-investigators/collaborators who provide trial specific expertise. This group will have responsibility for the day to day operational management of the trial. It will meet face to face or by teleconference on a monthly basis and will communicate between times via telephone and email as needed. The roles and responsibilities of the TMG will be detailed in the Trial Management Group Charter. Meetings will be formally minuted and stored in the Trial Master File (TMF).

Trial Steering Committee (TSC)

The conduct of the trial will be overseen by a Trial Steering Committee (TSC) on behalf of the Sponsor/Funder. The TSC will include the Chief Investigator (CI), 2 of the co-investigators and a group of experienced critical care clinicians and trialists as well as a "lay" representative. Regular meetings will be held, however as the Data Monitoring and Ethics Committee (DMEC) will meet to assess the accumulating data, the TSC may be convened to discuss issues and recommendations raised by the DMEC. The roles and responsibilities of the TSC will be detailed in the Trial Steering Committee Charter. The TSC, in the development of this protocol and throughout the trial, will take responsibility for monitoring and guiding overall progress, scientific standards, operational delivery and protecting the rights and safety of trial participants. Meetings will be formally minuted and stored in the Trial Master File (TMF).

Data Monitoring and Ethics Committee (DMEC)

A Data Monitoring and Ethics Committee (DMEC) will be appointed comprising two clinicians with experience in undertaking clinical trials / caring for critically ill patients / cell therapy and a statistician who are independent of the trial. The DMEC will meet to agree conduct and remit, and the roles and responsibilities of the DMEC will be detailed in the Data Monitoring and Ethics Committee Charter. The DMEC will be convened after each dose cohort of patients have been recruited into the phase 1 trial and completed at least 7 days follow-up to approve dose escalation as per the dose escalation plan. When 7 days of follow-up data for all study subjects in the phase 1 study are available the TMG will review the data and propose a cell dose for the phase 2 trial. This recommendation will be submitted to the DMEC for approval prior to initiating the phase 2 trial. In the phase 2 trial the DMEC will be convened monthly initially. Frequency of DMEC meetings should be kept under review by the DMEC and TSC. In the event of any safety concerns, additional unplanned DMEC meetings will be convened.

The DMEC's responsibility is to safeguard the interests of the trial participants, in particular with regard to safety, and assist and advise the TSC so as to protect the validity and credibility of the trial. The DMEC will monitor recruitment, adverse events and outcome data. During the recruitment period, reports will be provided to the DMEC which will include information on recruitment, AEs reported, and deaths from all causes at 28 and 90 days, along with any other data that the committee may request. The DMEC will advise the TSC on continuation of the trial. They will make recommendations to stop the trial for benefit on the basis of a sufficiently statistically significant benefit and an effect estimate that is sufficiently large to be likely to influence decisions about the use of the relevant therapy by clinicians outside of the trial. Meetings will be formally minuted and stored in the Trial Master File (TMF).

Following a recommendation from the DMEC, the TSC will decide what actions, if any, are required. It will be the responsibility of the TSC to inform the Sponsor if concerns exist about patient safety, following which the Sponsor will take appropriate action.

User Involvement or any Other Relevant Committees

Patient experience whilst critically ill will be taken into consideration when preparing patient information leaflets and consent forms. Barry Williams (previous Chairman of the Critical Care Patient Liaison Committee (CritPal); now known as PatRel) will represent the patient's perspective on the TSC ensuring that the trial remains considerate of the needs of the patients and their families.

4 BACKGROUND AND RATIONALE

4.1 Acute Respiratory Distress Syndrome

Acute Respiratory Distress Syndrome (ARDS) is a common condition of acute hypoxemic respiratory failure resulting from disruption of the alveolar-capillary barrier, and a resultant inflammatory pulmonary oedema. It can occur in the critically ill in response to many insults including severe trauma, burns, infection, major surgery or neurological disorders. ARDS is a leading cause of death and disability in critically ill adults and children worldwide (1), with a hospital mortality of approximately 40% (1-3). UK data suggest that up to 20,000 cases of ARDS occur annually; 50% of these fall into the “moderate -severe” category of ARDS and have up to 40% mortality (2, 4). ARDS confers a considerable long-term illness and disability burden on the individual sufferer and on society. Only 50% of survivors are able to return to work 12 months after hospital discharge, while cognitive, psychologic, and physical morbidity persists for up to 5 years (5, 6).

ARDS has significant resource implications, prolonging intensive care unit (ICU) and hospital stay, and requiring rehabilitation in the community, all of which impact on NHS services. Patients with ARDS account for 10% of intensive care unit (ICU) admissions, and because they have a long average stay in ICU they use up to a quarter of ICU bed-days (7). The high incidence, mortality, long-term consequences and high economic costs make ARDS a major clinical problem. The estimated global annual cost of ARDS is $613 million (USD). Within the NHS the estimated costs per patient up to 28 days are £22.5K and at 6 months are £30.5K (8).

There are no treatments for ARDS, and management remains supportive with lung-protective ventilation and restrictive fluid management (9). Among patients who receive low tidal–volume ventilation, mortality rates remain unchanged over the past two decades (10, 11) highlighting the need for novel therapeutic strategies. Unsuccessful large scale clinical trials of multiple therapeutic strategies, including nitric oxide (12, 13), anti-oxidants (14), surfactants (15), corticosteroids (16) and immunomodulating agents such as IL-10, GM-CSF, neutrophil elastase inhibitor (17), and high frequency oscillatory ventilation (18, 19) highlight the need for novel approaches for these patients. Most recently, statin therapy was found to be ineffective in 2 large trials, including the HARP-2 study of simvastatin (4). Therefore, innovative therapies are needed to reduce both the mortality associated with ARDS and the long-term morbidity in survivors and their carers. A treatment intervention that could improve outcome from ARDS, would have a major impact on patients, carers and NHS and Social Care resources.

4.2 ARDS in COVID 19

Current (20 March 2020) data on novel coronavirus disease (COVID-19) suggests that it carries a mortality rate of ~3.4%, compared with <0.1% with seasonal influenza.(20) ARDS occurs in approximately 20% cases of COVID-19 and respiratory failure is the leading cause of mortality.(21) In a retrospective multi-centre study of 150 confirmed cases in Wuhan, China, ARDS occurred in a significantly greater proportion of non-survivors 81% (55/68 patients) compared with only 9% of survivors (7/82 patients); p<0.01.(21) In another study of 193 confirmed COVID-19 cases, ARDS was observed at a significantly higher rate in non-survivors 93% (50/54 patients) compared with survivors, 7% (9/137 patients); p<0.0001.(22)

4.3 Mesenchymal Stromal Cells

Mesenchymal Stem Cells (MSCs) are a mononuclear cell population that, when cultured ex vivo, adhere to plastic with a fibroblast-like morphology (23), generate colony forming unit fibroblasts (CFU-F) in culture, and have the potential to differentiate into multiple lineages, and bone, cartilage and adipocyte cells in particular (24, 25). Although bone marrow is the most

often used source of MSCs, MSCs with similar biological properties have also been isolated from other tissues including adipose tissue, skeletal muscle and cord blood (26-29). Of special interest is umbilical cord, since it represents an abundant and accessible source of MSCs (29).

As this therapy has never been used in humans, there are no known risks. One of the defining properties of MSCs is their ability to undergo self- renewal and expansion. Because of these inherent cellular properties there is some concern that MSCs themselves have either the potential to undergo malignant change or to enhance the proliferation of malignant cells. Whilst there have been in vivo studies showing that systemically injected murine MSCs result in the formation of osteosarcomas due to karyotype abnormalities (30), this does not occur with human MSCs. To date MSCs have been used in many clinical trials for treatment of a wide range of diseases and there have been no long term adverse events including cancer reported (31).

Cell-based therapies have been termed the “next pillar of Medicine” (32). It is recognized that the capacity of cell based therapies to engraft, secrete paracrine factors or produce microparticles (microvesicles) or connect with other cells for mitochondrial exchange, all represent key potential advances in modulating tissue microenvironments, reducing inflammation and promoting repair in inflammatory or degenerative disorders (33-36). Mesenchymal Stem Cells (MSCs) constitute an innovative approach with substantial therapeutic promise for ARDS. They possess several favorable biological characteristics, including their convenient isolation, ease of expansion in culture while maintaining genetic stability (37), minimal immunogenicity and feasibility for allogenic transplantation (38).

4.4 Pre-clinical evidence for MSC efficacy and safety in ARDS

MSCs offer considerable promise as a novel therapeutic strategy for ARDS: MSCs reduce inflammation and enhance bacterial clearance during rodent and murine bacterial pneumonia (33-36), and augment repair of the animal (39, 40) and human lung (41).

Large animal studies have also replicated these beneficial effects. Bone marrow derived (BM) hMSCs decreased acute lung injury (ALI), without producing organ toxicity, in endotoxin injured sheep (42). We tested the effects of human bone marrow-derived plastic adherent MSC in a clinically relevant large animal (ovine) model of ARDS induced by smoke inhalation and Pseudomonas aeruginosa (43). Sheep were randomised to receive one of two doses of MSCs (5 or $10x10^6$ cells/kg) or identical control, 1 hour following injury. A single dose of intravenous MSCs therapy caused a significant 3-fold improvement in oxygenation without adverse haemodynamic or respiratory events (43).

hMSCs have been studied in animal models of viral induced lung injury. BM derived hMSCs in aged mice in a H5NI viral model increased survival, reduced evidence of lung injury, reduced pro-inflammatory cytokines and lung viral titres.(44) Umbilical cord (UC) derived hMSCs in a mouse H5N1 viral model restored alveolar fluid clearance and decreased BAL inflammatory cytokines. (45)

4.5 Clinical evidence for MSC safety and efficacy in ARDS

Two randomised small phase 1 studies of plastic adherent MSCs in patients with ARDS have taken place. In China, investigators used adipose-derived plastic adherent cells in a small cohort (n=12) of patients with ARDS randomised 1:1 to MSCs or placebo (46): they showed that the cells were safe and well-tolerated in this patient group, and were associated with reduced plasma levels of the alveolar epithelial cell injury marker SP-D. In the US our co-investigator, Matthay, has completed the phase 1 START trial, using a dose escalation study of plastic adherent bone marrow derived MSCs, in patients with moderate to severe ARDS.

START showed that marrow derived MSCs at similar doses to those proposed in our study are safe and well-tolerated, (n=9) (47).

During the current COVID-19 pandemic, MSCs have been administered in a pilot study of seven patients with COVID 19 pneumonia.(48) A single infusion of 1 x 10^6 MSCs were administered intravenously when their condition was considered to be deteriorating despite other treatments however only 1 of the 7 patients required critical care and mechanical ventilation. 4 patients were described as severe with compromise of respiratory function. Patients underwent follow up for 14 days and no infusional toxicity or adverse events were reported.

4.6 Dose regimen rationale

The dose range for the Phase 1 study and the choice of a single infusion were based on available pre-clinical data. In a rodent model of E. coli induced ARDS (33) a dose-range was studied (2, 5, 10 million cells/kg). Efficacy, as determined by arterial oxygenation, inflammatory cytokine reduction and bacterial clearance was demonstrated at 5 and 10 million cells/kg.

In a clinically relevant ovine model of ARDS induced by smoke inhalation and Pseudomonas aeruginosa, sheep were randomised to receive one of two doses of human bone marrow-derived plastic adherent MSCs (5 or 10x10^6 cells/kg) reconstituted in Plasma-Lyte or identical Plasma-Lyte A control 1 hour following injury. A single dose of intravenous MSCs caused a significant 3-fold improvement in oxygenation without adverse haemodynamic or respiratory events. The P/F ratio was similar in groups treated with 5 or 10x10^6 cells/kg (43). In a pig animal model of oleic acid induced lung injury, infusion of 2x10^6cells/kg reduced lung tissue nuclear NFkB translocation (49).

In the phase 1 START trial in patients with moderate to severe ARDS a dose-range of 1, 5 and 10 million cells/kg was studied. Although a small study, there was no significant difference in terms of efficacy between the doses in surrogate clinical outcomes [42]. In a human study of diabetic nephropathy, 150 but not 300 million MSCs improved renal function (50). Similarly in a study of patients with degenerative spinal disc disease, a single injection of 6million MSCs was more efficacious than 18milllion cells (51).
There are theoretical concerns that at very high doses coagulation markers on the MSC surface may be triggered, leading to loss of cells and activation of coagulation system in recipients. These effects have not been identified in patients treated with the dose range 1-3x10^6 cells/kg (52).

We have chosen to initially assess the safety of Orbcel-C over a dose-range of 1-6 million cells/kg, a dose range compatible with most previous human clinical trials of MSCs. To simplify cell manufacture and delivery of the intervention we have chosen 100, 200 and 400 million cells which equates range of approx. 1.4, 2.8 and 5.7 million cells/kg for predicted body weight of 70kg.

These data have informed the use of a single infusion of MSCs as well as the dose planned in the phase 2 study. We have chosen intravenous administration as this is a route we have seen efficacy with in pre-clinical studies and because this is the route used in the vast majority of clinical studies.

4.7 Immunologic monitoring

Previous studies have indicated MSCs are relatively immune-privileged, however pre-clinical and clinical studies have reported the development of anti-HLA antibodies in both animal models (53) and humans (54-56) following MSC therapy. As a result, a potential issue is that patients forming anti-HLA antibodies may face added risk if subsequently they undergo organ

transplantation. The clinical impact of anti-HLA antibodies is not clear for patients undergoing transplantation. Furthermore ARDS is an acute rather than chronic condition and it is unlikely to be followed by the need for solid organ transplantation. Therefore, the clinical impact of anti-HLA antibodies is likely to be very low in this context. Nevertheless, should patients develop high titre anti-HLA antibodies, as reported by the Tissue Typing laboratory, the treating clinician and patient will be informed of the finding. In the unlikely event that there should be a clinical concern, referral to Immunology services will be arranged.

4.8 Rationale for a trial of REALIST ORBCEL-C in ARDS

CD362 enriched umbilical cord-derived MSCs (REALIST ORBCEL-C) have been developed to maximise purity and efficacy, to address the new requirements likely to be imposed on ATMPs (Advanced Therapeutic Medicinal Products) by both European and British regulators, and to maximise availability of cells for cost-effective delivery within the NHS and other larger patient populations. By using umbilical cord rather than bone marrow as the source material for the MSCs, the costs of the cellular product (by avoiding need for marrow donation, surgical procedures & theatre time), are markedly reduced, which has implications for the health economic benefits. Umbilical cord tissue is routinely discarded after cord blood donations (10,000 donations/year in UK alone), so this maximises potential donations from what is otherwise a waste product. The cellular product is consistent in age in terms of donor, and umbilical cord yields a greater increase in cells after in vitro cell expansion. A process for procuring cord tissue under HTA license is established by the NHSBT.

Currently, clinical doses of plastic adherent MSCs are isolated by plating dissociated tissue onto tissue culture plastic and sub-culturing over one or more passages. Recent EU (CAT/571134) and British Standard Institute (PAS-93) documents indicate that the plating method is inadequate for defining or purifying MSCs for cell manufacture as an ATMP for clinical use. For example, only approximately 1 in 50,000 of bone marrow mononuclear cells (BM MNCs) plated are actually MSC. Both documents note a requirement for more markers to define and prospectively isolate cells for therapeutic use. Using CD362mAb for isolation from either marrow or umbilical cord markedly increases the MSC/MNC purity ratio to 1 in 3. Human CD362 enriched MSC have been independently tested and displayed efficacy in pre-clinical models of disease including murine models of diabetic nephropathy, neuropathy, retinopathy, cardiomyopathy and liver inflammation. In addition, CD362 enriched MSC have improved graft survival in a rat model of cornea transplant rejection. CD362 enriched MSC improve wound closure in a rabbit model of diabetic ulcers (57). Finally, CD362 enriched MSC improve arterial oxygen, reduce inflammation in both ventilator-induced and E. coli-induced rat models of acute lung injury (unpublished data).

5 STUDY AIM AND OBJECTIVES

5.1 Research Hypothesis

In young people (aged 16-17 years) and adult patients with moderate to severe ARDS, human umbilical cord derived CD362 enriched MSCs, (REALIST ORBCEL-C cells) are safe and improve important outcomes.

5.2 Study Aim

The aim of this study is to conduct a phase 1 and a phase 2 clinical trial of human umbilical cord derived CD362 enriched MSCs, (REALIST ORBCEL-C cells), in patients with ARDS.

5.3 Study Objectives

Primary objective

To assess the safety of a single intravenous infusion of REALIST ORBCEL-C cells in patients with ARDS.

Secondary objectives

In patients with moderate to severe ARDS to determine the effect of a single intravenous infusion of REALIST ORBCEL-C cells on:

1. Physiological indices of respiratory dysfunction reflecting severity of ARDS, as measured by oxygenation index (OI), respiratory compliance, and P/F ratio.
2. Sequential organ failure assessment (SOFA) score.
3. Extubation and reintubation.
4. Ventilation free days at day 28.
5. Duration of ventilation.
6. Length of ICU and hospital stay.
7. 28-day and 90-day mortality.

6 STUDY DESIGN

6.1 Study Design

The phase 1 trial is an open label dose escalation pilot study in which cohorts of subjects with moderate to severe ARDS will receive increasing doses of a single infusion of REALIST ORBCEL-C in a 3+3 design (Figure 1). We initially plan 3 cohorts with 3 subjects/cohort. Planned doses for the 3 cohorts pending absence of safety concerns are 100 x 10^6 cells, 200 x 10^6 cells and 400 x 10^6 cells. (Figure 2).

In the completed Phase 1 REALIST trial, infusion of 400 million cells was achieved without any dose limiting toxicity at day 7 and has been approved by the DMEC as the intervention dose for this study.

The phase 2 trial is a randomised, double-blind, allocation concealed placebo-controlled study of 400 x10^6 cell dose of REALIST ORBCEL-C in patients with moderate to severe ARDS (Figure 1). The phase 2 trial will recruit patients with ARDS due to COVID-19.

In PICO terms:

Population	Young people (aged 16-17 years) and adult patients with moderate to severe ARDS due to COVID-19
Intervention	400x10^6 cell dose of REALIST ORBCEL-C
Comparator	Placebo
Outcome	Safety and physiological indices of efficacy

6.2 Study Schematic Diagrams

Figure 1: Flow diagram for the phase 1 trial

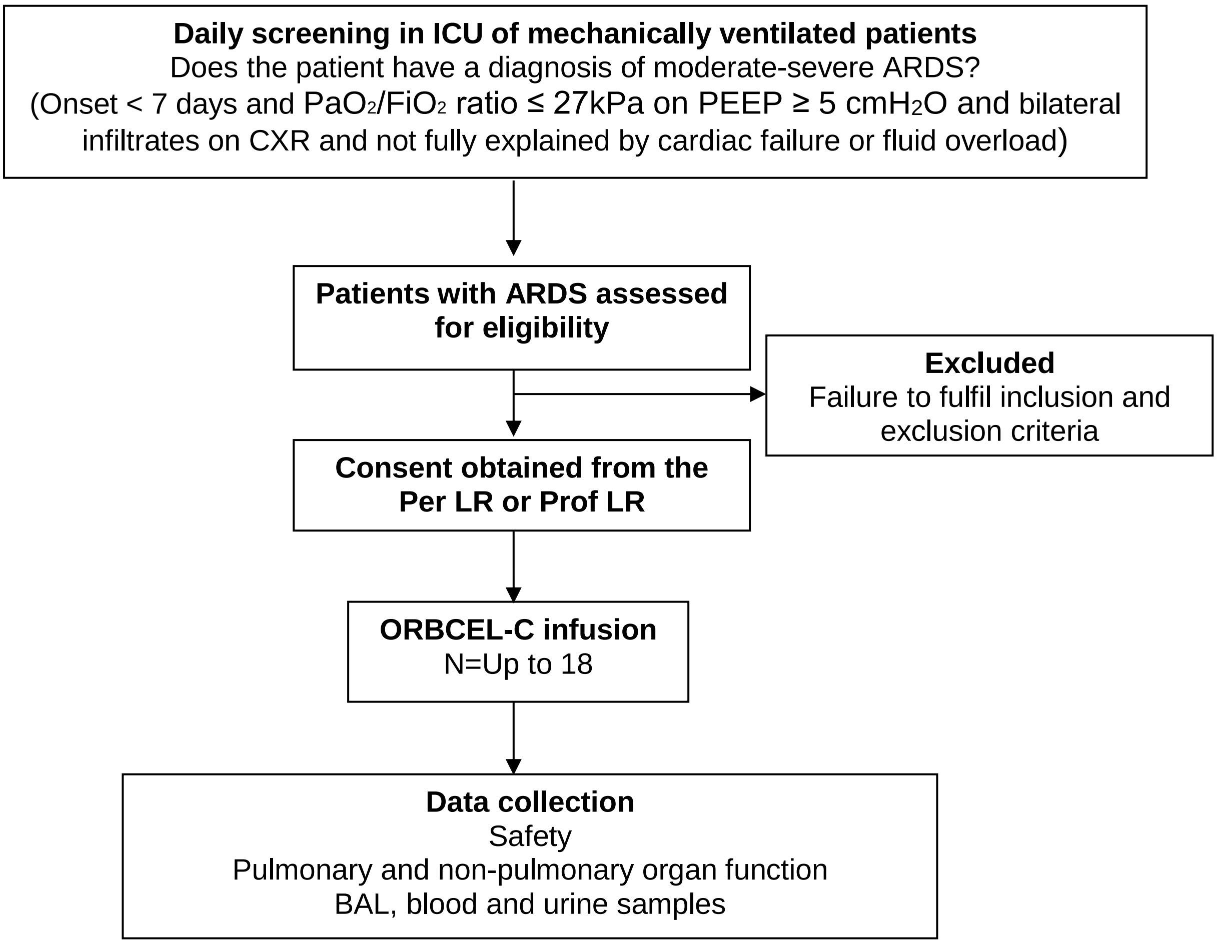

Figure 2: Flow diagram for the 3+3 trial design to determine the maximal tolerated dose (MTD

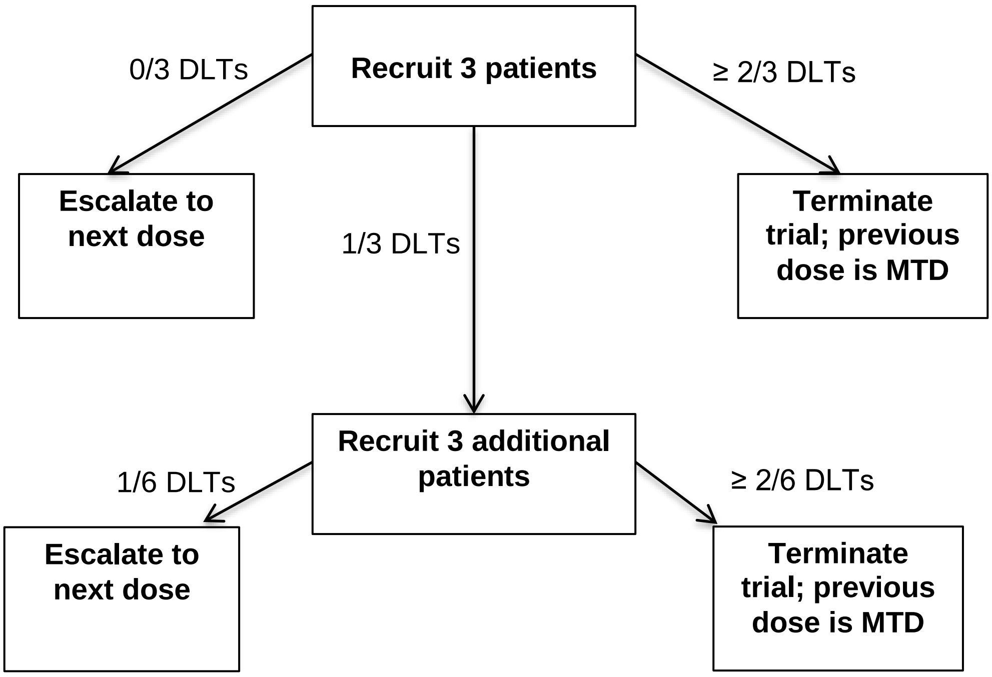

Patients will be assessed to day 7 for dose limiting toxicity (DLT) to determine dose escalation. The maximum dose will be 400 x 10^6 cells.

Figure 13: CONSORT flow diagram for trial conduct for the phase 2 trial

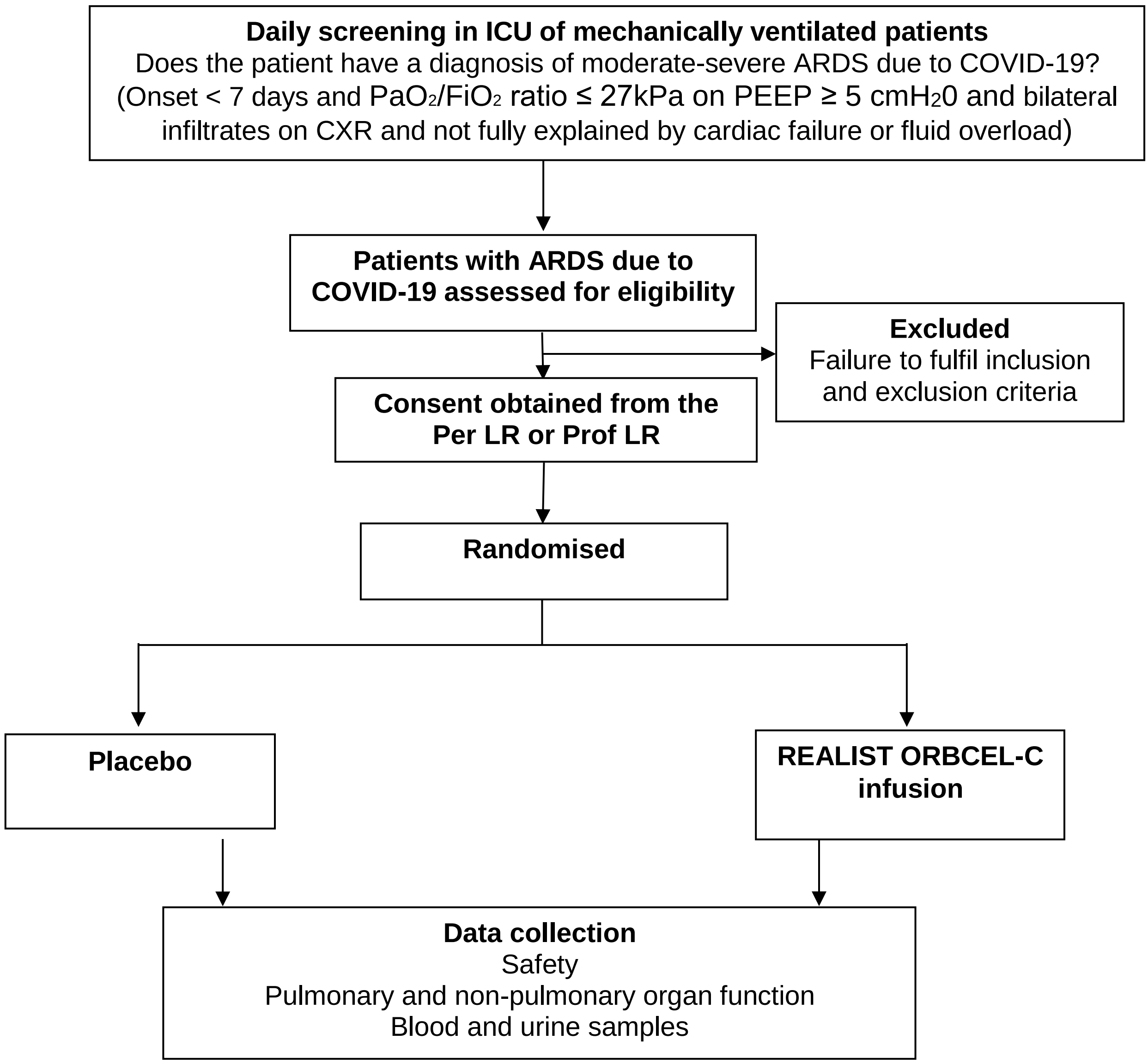

6.3 Study Timeline

The overall duration of the study is 60 months. Recruitment to the phase 2 study will occur during the COVID-19 pandemic. Patients will be followed up until 2 years post study drug administration.

6.4 End of Study

The trial will end when all phase 1 and phase 2 patients have completed up to 2 year follow-up and database lock occurs.

The trial will be stopped prematurely if:

- Mandated by the Research Ethics Committee (REC)
- Mandated by the Medicines and Healthcare products Regulatory Agency (MHRA)
- Mandated by the Sponsor e.g. following recommendations from the DMEC
- Funding ceases

The REC that originally gave a favourable opinion of the trial and the MHRA who issued the clinical trial authorisation (CTA) will be notified in writing if the trial has been concluded or terminated early.

.

7 STUDY OUTCOME MEASURES

The phase 2 study will evaluate safety and efficacy outcomes in patients with ARDS due to COVID-19 who have been administered MSC therapy. Although the primary focus of the phase 2 trial is safety, several outcomes will be evaluated to determine whether treatment with MSCs shows efficacy for important outcomes in patients with ARDS due to COVID-19.

7.1 Primary Outcome Measure

The primary safety outcome is the incidence of serious adverse events (SAEs).

The primary efficacy outcome is oxygenation index (OI) at day 7.

OI is a physiological index of the severity of ARDS and measures both impaired oxygenation and the amount of mechanical ventilation delivered. OI is independently predictive of mortality in patients with ARDS (58, 59). We have chosen day 7 as we expect this time interval will minimise the competing effects of death and extubation, while allowing a sufficient time interval for a biological effect to occur.

OI is calculated as (mean airway pressure (cm H_2O) x FiO2 x 100) ÷ PaO_2 (kPa). These simple measurements are easily and routinely collected as part of standard ventilator practice.

7.2 Secondary Outcome Measures

The following clinical outcomes will also be assessed:

1. OI at days 4 and 14.
2. Physiological indices of ARDS, as measured by respiratory compliance (Crs), driving pressure and P/F ratio on days 4, 7 and 14.
3. Organ failure as measured by the sequential organ failure assessment (SOFA) score on days 4, 7 and 14.
4. Extubation and reintubation.
5. Ventilation free days at day 28.
6. Duration of ventilation.
7. Length of ICU and hospital stay.
8. 28-day and 90-day mortality.

Outcomes will be measured at baseline and daily up to day 14 or until the patient is discharged from ICU or the patient dies, and thereafter as necessary for the 28-day and 90-day mortality.

Patients will be followed up annually up to 2 years following study drug administration.

8 PATIENT ELIGIBILITY

8.1 Study Setting

The main trial will take place in ICUs that are able to care for adult level 3 patients as previously defined (60). Sites will be chosen on the basis of experience in clinical trials of investigational medicinal products in ARDS and a track record in successfully recruiting patients to such studies. Staff must also demonstrate and document a willingness to comply with the protocol, standard operating procedures (SOPs), the principles of GCP (Good Clinical Practice), regulatory requirements and be prepared to participate in training. A training package will be

provided to sites who participate in the study. A list of study sites will be maintained in the TMF.

8.2 Study Population

Patients will be prospectively screened daily. During the phase 1 trial, all patients with moderate to severe ARDS will be entered into a screening log. If possible during the phase 2 trial, all patients with moderate to severe ARDS due to COVID-19 will be entered into a screening log. If the patient is not recruited the reason will be recorded. A fully anonymised minimal dataset will be recorded on these patients (age, gender, APACHE II score, worst P/F ratio at time of assessment, reasons for non-enrolment and vital status). APACHE II score and vital status will be collected using anonymised linkage to the ICNARC database through a defined CMP number (or equivalent). This will allow comparison to identify that the study population is representative of the overall cohort of patients. This information is required to establish an unbiased study population and to ensure the study can be reported in keeping with CONSORT guidelines (www.consort-statement.org).

8.3 Eligibility Criteria

Eligibility to participate in the trial will be confirmed by a medically qualified person who is named on the delegation log. The medical care given to, and medical decisions made on behalf of subjects, will be the responsibility of an appropriately qualified treating physician.

The P/F ratio table in appendix 1 can be used for reference. Patients will be eligible to participate in the study if they fulfil the following criteria:

Inclusion criteria

1. Moderate to severe ARDS as defined by the Berlin definition (61).
   a) Onset within 1 week of identified insult
   b) Within the same 24-hour time period
   i. Hypoxic respiratory failure (PaO_2/ FiO_2 ratio ≤ 27kPa on PEEP ≥ 5 cmH_20)
   ii. Bilateral infiltrates on chest X-ray consistent with pulmonary oedema not explained by another pulmonary pathology
   iii. Respiratory failure not fully explained by cardiac failure or fluid overload
   The time of onset of ARDS is when the last ARDS criterion is met.
2. Patient is receiving invasive mechanical ventilation.
3. COVID-19 based on clinical diagnosis or PCR test.

Exclusion criteria

1. More than 72 hours from the onset of ARDS.
2. Age < 16 years.
3. Patient is known to be pregnant.
4. Major trauma in the prior 5 days.
5. Presence of any active malignancy (other than non-melanoma skin cancer) that required treatment within the last year.
6. WHO Class III or IV pulmonary hypertension.
7. Venous thromboembolism currently receiving anti-coagulation or within the past 3 months.
8. Currently receiving extracorporeal life support (ECLS).
9. Severe chronic liver disease with Child-Pugh score > 12.
10. DNAR (Do Not Attempt Resuscitation) order in place.
11. Treatment withdrawal imminent within 24 hours.
12. Consent declined.

13. Prisoners.
14. Non-English speaking patients or those who do not adequately understand verbal or written information unless an interpreter is available.
15. Previously enrolled in the REALIST trial.

Our inclusion and exclusion criteria are designed to include those who reflect the general population of critically ill patients with COVID-19 ARDS who may benefit from the therapeutic intervention and exclude patients who may be more likely to experience an adverse reaction.

A pregnancy test in females with child bearing potential (aged 15-55 years) will be performed prior to enrolment and patients who are pregnant will be excluded. Given the population being recruited is critically ill the need for contraception advice is recognised to be very unlikely. At the discretion of the investigator, contraception advice will be given to patients at hospital discharge who may be sexually active prior to day 90.

8.4 Co-enrolment guidelines

Patients in the REALIST study are potentially eligible for co-enrolment in other non-CTIMP studies, this will be decided on a case by case basis in keeping with UK guidelines for critical care research (62). The Clinical Trials Unit (CTU) should be informed if co-enrolment occurs. Co-enrolment with any studies should be documented in the CRF.

9 PATIENT SCREENING, CONSENT and RECRUITMENT

9.1 Patient Screening

All mechanically ventilated patients in the ICU will be screened daily each morning for eligibility. Patients clinically judged to have hypoxaemic respiratory failure will be screened against the inclusion and exclusion criteria. Eligible patients will then be discussed with their treating ICU physician to confirm their agreement with trial enrolment.

9.2 Informed Consent Procedure

The study will be conducted in accordance with the ethical principles that have their origin in the Declaration of Helsinki. The Principal Investigator (PI) (or designee) is responsible for ensuring that informed consent for trial participation is given by each patient or a legal representative. An appropriately trained doctor or nurse may take consent. The person taking informed consent must be GCP trained, suitably qualified and experienced and have been delegated this duty on the delegation log. Appropriate signatures and dates must be obtained on the informed consent documentation prior to collection of trial data and administration of the trial drug. If no consent is given a patient cannot be enrolled into the trial.

The incapacitating nature of the condition precludes obtaining prospective informed consent from participants. In this situation informed consent will be sought from a Personal Legal Representative (Per LR) or Professional Legal Representative (Prof LR).

Personal Legal Representative Consent

Informed consent will be sought from the patient’s personal legal representative (PerLR) who may be a relative, partner or close friend. The PerLR will be informed about the trial by the responsible clinician or a member of the research team and provided with a copy of the covering statement for the PerLR with an attached participant information sheet (PIS) and asked to give an opinion as to whether the patient would object to taking part in such medical research. If the PerLR decides that the patient would have no objection to participating in the

trial they will be asked to sign the PerLR consent form which will then be countersigned by the person taking consent. The original will be retained in the investigator site file (ISF) and a copy given to the PerLR and another copy placed in the patients' medical records.

During the COVID-19 pandemic, there are likely to be visiting restrictions in place due to infection control measures and therefore it may not be possible to obtain consent from the PerLR at the clinical site. If the PerLR is not available at site, the researcher may contact the PerLR by telephone and seek verbal agreement. This verbal agreement will be recorded in the PerLR Telephone Agreement Form. The PerLR Telephone Agreement Form will be signed by a second member of staff who has witnessed the telephone advice. This witness may be a member of the site study team or site medical staff. A copy of the PerLR Telephone Agreement Form should be placed in the patient's medical notes and a copy filed in the ISF. Written consent will then be obtained if possible.

Professional Legal Representative Consent

As the patient is unable to give informed consent and if no PerLR is available, a doctor who is not connected with the conduct of the trial may act as a professional legal representative (ProfLR). The doctor will be informed about the trial by the responsible clinician or a member of the research team and given a copy of the PIS. If the doctor decides that the patient is suitable for entry into the trial they will be asked to sign the professional legal representative consent form. The original will be retained in the investigator site file (ISF) and a copy given to the ProfLR and another copy placed in the patients' medical records.

Retrospective Patient Consent

Patients will be informed of their participation in the trial by the responsible clinician or a member of the research team once they regain capacity to understand the details of the trial. The responsible clinician or a member of the research team will discuss the study with the patient and the patient will be given a copy of the PIS to keep. The patient will be asked for consent to continue to participate in the trial and to sign the consent to continue form which will then be countersigned by the person taking consent. The original will be retained in the investigator site file (ISF) and a copy given to the patient and another copy placed in the patients' medical records. Where consent to continue is not obtained, consent from the legal representative will remain valid. If the patient refuses consent, permission to use data collected to that point and to access medical records for trial data will be requested from the patient.

Withdrawal of Consent

Patients may withdraw or be withdrawn (by PerLR or ProfLR) from the trial at any time without prejudice. In the event of a request to withdraw from the study, the researcher will determine which elements of the trial are to be withdrawn from the following possibilities and this will be documented:

- REALIST ORBCEL-C administration if ongoing
- On-going data collection during hospital admission
- On-going data collection following hospital discharge
- Confirmation of vital status

In the event that the request is to withdraw from all elements of the study, only anonymised data recorded up to the point of withdrawal will be included in the study analysis. Consent will also be requested to use the samples collected to that point. Similar consent mechanisms have been used successfully in other critical care trials (4, 8, 9, 63, 64).

10 ASSIGNMENT OF INTERVENTION

10.1 Allocation to the Phase 1 Trial

Participants will be allocated to receive either 100, 200 or 400 x 10^6 dose of REALIST ORBCEL-C.

After informed consent, patients will be allocated to the appropriate dose cohort via the CTU. Sites will be provided with trial specific allocation guidelines. Allocation will be completed by an appropriately trained and delegated member of the trial team. At the time of dose allocation, each patient will be allocated a unique Participant Study Number, which will be used throughout the study for participant identification. An entry will be recorded in the patients' medical notes noting enrolment into the study.

The CTU will manage the recruitment process to confirm when a contacting site can enrol a patient to a dose cohort to ensure only one patient across all sites is treated at a time and that each patient will receive treatment no less than 24 hours following the completion of treatment of another patient.

A dose escalation plan will be followed. Once the necessary number of patients have been recruited to a dose cohort the CTU will communicate to sites and not allow further patients to be allocated treatment until the DMEC has approved escalation to the next dose. An email will be sent to all site PIs when a dose cohort has been completed and when the DMEC has approved escalation to the next dose.

10.2 Randomisation for the Phase 2 Trial

Participants will be allocated to REALIST ORBCEL-C or placebo control in a 1:1 ratio. Randomisation will be stratified by recruitment centre and vasopressor use.

After informed consent, patients will be randomised via a centralised randomisation system. Sites will be provided with trial specific randomisation guidelines. Randomisation will be completed by an appropriately trained and delegated member of the research team. The randomisation sequence will be saved in a restricted section of the TMF, which can only be accessed by the trial statistician and not those who enrol or assign interventions. At the time of randomisation, each patient will be allocated a unique Participant Study Number, which will be used throughout the study for participant identification. An entry will be recorded in the patients' medical notes noting enrolment into the study.

10.3 Blinding

The cell therapy facility and clinical trials pharmacist will be unblinded. The unblinded individuals will keep the treatment information confidential and will not discuss or release information on treatment allocation to the patient, the investigator, or other members of the research team.

As in prior studies of MSCs (47, 65), the infusion bag containing either the cell product or placebo will be masked at the time of preparation in the clinical site's cell therapy facility so that the contents of the infusion bag are not visible to the investigators or to the clinicians who are administering the study drug. The contents of the infusion bag will be administered through a masked infusion set.

10.4 Unblinding Procedure

The investigator or treating physician may unblind a participant's treatment assignment in the case of an emergency, when knowledge of the study treatment is essential for the appropriate clinical management or welfare of the patient. Should a treating clinician require emergency unblinding, they should contact the centralised allocation system and follow the trial specific unblinding guidelines. Unblinding will generate an email alert to the Trial Manager and CI. The date and reason for the unblinding must be recorded in the CRF.

11 INVESTIGATIONAL MEDICINAL PRODUCT

The Investigational Medicinal Products are:

a. Allogeneic donor CD362 enriched human umbilical cord-derived mesenchymal stromal cells (REALIST ORBCEL-C) supplied as a sterile, single-use cryopreserved cell suspension containing a fixed cell dose of either 100 x 10^6, 200 x 10^6 or 400 x 10^6 cells in 10mL, 20mL or 40mL volumes, respectively to be further diluted in Plasma-Lyte 148 to a total volume of 200mls for the purposes of administration.

b. Plasma-Lyte 148 Solution for Infusion (200mls) as the placebo control solution.

Further information on REALIST ORBCEL-C and Plasma-Lyte 148 (placebo control) can be obtained from the Investigational Medicinal Product Dossier (IMPD).

All patients will be administered chlorphenamine 10mg (which is regarded as a Non-Investigational Medicinal Product for the purposes of this trial) by peripheral IV bolus, to be given prior to infusion. This will be supplied from hospital commercial stock. Chlorphenamine 10mg is licensed for the control of allergic reactions and will be supplied directly from hospital pharmacy.

11.1 Study Intervention

***Table 1: Trial intervention in "TIDieR" format** (66)*

TIDieR item number	Item descriptor	Item
1	Brief name	Allogeneic donor CD362 enriched human umbilical cord-derived mesenchymal stromal cells (REALIST ORBCEL-C)
2	Why	Allogeneic mesenchymal stromal cells (MSCs) have multiple actions which have the potential to reduce inflammation, promote repair and reduce infection in ARDS.
3	What materials	The investigational cellular product will be allogeneic donor CD362 enriched human umbilical cord-derived Mesenchymal Stromal Cells (REALIST ORBCEL-C)<br><br>The investigational product is harvested from umbilical cords of unrelated and human leukocyte antigen (HLA) unmatched healthy donors, collected and manufactured to GMP level by Cellular Molecular Therapies at the National Health Service Blood and Transplant (NHSBT) group.<br><br>The cellular product is cryopreserved in Cryostor CS10 for long-term storage. The MSCs will be stored under controlled conditions in the vapour phase of liquid nitrogen tanks. The MSCs will be transported using validated shippers to the clinical site's cell therapy facility.<br>The Placebo will be Plasma-Lyte 148 solution for infusion.

4	What procedures	Patients will be pre-treated with 10mg intravenous chlorphenamine, after which a single dose of REALIST ORBCEL-C or placebo (Plasma-Lyte 148) will be administered intravenously over approximately 30 to 90 minutes.
5	Who provides	An appropriately trained clinician will administer the infusion according to the IMP administration guidelines. A member of the research team will be available for safety monitoring during the infusion of REALIST ORBCEL-C or placebo and for 5 hours following the completion of the infusion.
6	How	The REALIST ORBCEL-C substance will be thawed and re-suspended in Plasma-Lyte 148 in the clinical site's cell therapy facility by the cell therapy facility staff according to the Product Specification.<br><br>The cell therapy facility or site pharmacy staff will be responsible for preparing the Plasma-Lyte 148 placebo.<br><br>The study drug infusion should be commenced as soon as possible after delivery to site ICU and infusion completed within 6 hours of the start of the thaw procedure.<br><br>A single dose of the study drug will be administered intravenously over approximately 30 to 90 minutes.<br><br>Only 1 patient will be treated at a time. For the phase 1 trial, the CTU will manage the recruitment process and will communicate to sites when a patient can be recruited to ensure only 1 patient across all sites is treated at a time and that each patient will receive treatment no less than 24 hours following the completion of treatment of another patient. This does not apply to the phase 2 trial.<br><br>A single dedicated infusion line should be used for cell administration. The study drug can be infused via peripheral or central venous access, though if administered through a peripheral intravenous access, it should be at least 20-gauge, and ideally 18-gauge. The cells are administered through a standard blood filter tubing set with a 170 to 260 micron filter.
7	Where	General adult ICUs
8	When and how much	For phase 1 and 2, the study drug will ideally be commenced within 12 hours of enrolment.<br>For the phase 1 study the dose escalation groups will be 100, 200 and 400 x 10^6 cells. 400 x10^6 cells has been determined as the dose for the phase 2 study.

9	Tailoring	During the completed phase 1 REALIST trial, three cohorts of three patients received escalating doses of 100, 200 and $400x10^6$ cells. No dose limiting toxicity occurred in any cohort and 400 x 10^6 cells was determined to be the maximum tolerated dose.
10	How well	Training will be delivered in each of the study sites. All sites will receive standardised training.

11.2 Study Drug Supply

National Health Service Blood and Transplant (NHSBT) will manufacture REALIST ORBCEL-C and distribute it to each of the cell therapy manufacturing facilities. The cell therapy manufacturing facilities will thaw and further dilute REALIST ORBCEL-C to produce the final infusion solution for administration to the patient. The cell therapy manufacturing facilities or site hospital pharmacy department will provide the Plasma-Lyte 148 placebo solution for infusion. The IMP will be labelled in accordance with regulatory requirements. Trial guidelines will provide detailed information regarding the protocol for storage, thawing, preparation of final infusion solutions, labelling and administration of REALIST ORBCEL-C or Plasma-Lyte 148 placebo solution.

11.3 Study Drug Accountability

The site clinical trials pharmacist and cell therapy facility staff will maintain full accountability for the study drug received, prepared and dispensed to patients in ICU. Records will be kept allowing traceability between the cell donor and the IMP recipient. Drug administration will be recorded on the patient's prescription chart.

11.4 Study Drug Infusion

The study drug infusion will be commenced as soon as possible following delivery to clinical site. Infusion should be completed prior to the expiry date/time stated on the product label.

The study drug administration guideline will provide detailed information on drug administration.

11.5 Study Drug Termination Criteria

Study drug will be discontinued if one of the following is met:

1. Study drug related adverse event.
2. Study drug expiry.
3. Death or discontinuation of active treatment.
4. Request from PerLR or ProLR to withdraw the patient from the study.
5. Decision by the attending clinician on safety grounds.

11.6 Study Drug Return and Destruction

Any partially used study drug should be disposed of/destroyed at the site in accordance with trial guidelines and local biological waste management policies.
Unused product should be disposed of/destroyed under the supervision of the site clinical trials pharmacist. Records of return and destruction will be maintained.

11.7 Concomitant Care

All aspects of intensive care will be according to standard critical care guidelines.

Mechanical Ventilation

Ventilation according to the ARDSNetwork ARMA trial, which demonstrated a reduction in mortality using a tidal volume of 6 ml/kg PBW will be the standard of care [9]. Target tidal volumes will be ≤ 6 ml/kg PBW to maintain a plateau pressure ≤ 30cmH2O. The recommended Positive End Expiratory Pressure (PEEP) will be based on the ARDSnet ARMA trial [9]. (See appendix 2)

Oxygenation will be titrated aiming for SpO_2 of 88%-95% or PaO_2 7-10kPa. Permissive hypercapnic respiratory acidosis aiming for a pH ≥ 7.2 is recommended.

Neuromuscular Blocking Drugs

Patients in both the intervention and control arm can receive neuromuscular blocking drugs (NMBD) at any stage to ensure patient-ventilator synchrony. This is in keeping with recent evidence that suggests NMBD are of benefit in early hypoxaemic respiratory failure due to ARDS [60].

Refractory Hypoxaemia

If the treating physician is concerned about hypoxaemia, interventions including prone positioning or referral for consideration of extracorporeal membrane oxygenation (ECMO) can be applied in either arm of the trial as per standard care in the UK.

Intravenous Fluid and Blood Therapy

It is recommended that patients will be managed with a conservative fluid balance strategy according to best evidence for patients with hypoxaemic respiratory failure (67). Blood transfusions should be in keeping with the best practice of a restrictive transfusion policy (68).

11.8 Management During the Study Drug Infusion

In keeping with standard care of critically ill patients in ICU, patients will be continuously monitored. Guidelines for the management of events during the study drug Infusion are provided in appendix 3.

12 SCHEDULE OF ASSESSMENTS

12.1 Schedule of Assessments

All patients recruited to the Phase 1 and Phase 2 of the trial must be evaluated according to the schedule of assessments as outlined in Table 2. Data will be collected at each of the following time points:

Table 2: Schedule of Assessments

	Day 0	Day 1	Day 2-3	Day 4	Day 5-6	Day 7	Day 8-13	Day 14	Day 15-28	Day 90 (+/- 14 days)	1 Year (+/- 30 days)	2 Year (+/- 30 days)
Eligibility assessment	X											
Informed consent	X											
Enrolment/ Randomisation	X											
Baseline data	X											
Daily data		X	X	X	X	X	X	X				
Chlorphenamine administration		X										
Study drug administration		X										
Adverse events		X	X	X	X	X	X	X	X	X		
ECHO data***	X			X								
Blood sampling*,**	X			X		X		X			X	X
Anti-HLA Ab%	X								X			
Urine sampling*,**	X			X		X		X				
Mortality$									X	X	X	X
Medical Event#											X	X

**Blood and urine samples for translational studies will be collected where possible
***Echocardiography data will be obtained where possible.
%Blood for anti-HLA Ab will be collected on day 0 and day 28 only where possible
$Mortality, including cause of death.
#Any significant medical event

12.2 Study Visits and Procedures

Day 0 (Baseline)

Baseline data (day 0) is the 24 hours preceding the time of recruitment which is defined as the time of study drug administration. If more than one value is available for this 24-hour period

the value closest but prior to the time of study drug administration will be recorded. Day 0 (baseline) data collected will include but is not limited to:

- Patient demographics
- Date of birth, gender, height, weight, PBW
- ICNARC Case Mix Programme (CMP) number or equivalent
- Date and time of ICU admission
- Date/time of onset of mechanical ventilation
- Date/time of consent and enrolment (phase 1) or randomisation (phase 2)
- Aetiology of ARDS
- The Acute Physiology And Chronic Health Evaluation score II (APACHE II)
- First qualifying P/F ratio (including date/time)
- Worst P/F ratio (including date/time)
- Murray Lung Injury Score
- Determinants of the SOFA score
- Temperature
- Ventilation parameters including but not limited to: Mode of ventilation, minute volume, RR, mean airway pressure, plateau pressure, PEEP
- Arterial blood gas including but not limited to FiO2, PaO2, PaCO2, pH, lactate
- Oxygenation Index
- Use of adjunctive therapies
- Renal replacement therapy
- Clinical laboratory assessments: renal, liver function, ferritin and CRP, haematological and coagulation parameters where possible
- Blood for anti-HLA antibodies will be collected where possible
- Echocardiography parameters (during phase 2 study only) including but not limited to ventricular size and function and tricuspid annular plane systolic excursion (TAPSE) will be collected where possible.
- Clinical or PCR diagnosis of COVID-19

Baseline blood and urine (where possible) will be taken prior to study drug administration. Sampling procedures are outlined below.

Day 1

Day 1 is from the time of recruitment (study drug administration) to the end of that calendar day. If more than one value is available for this period, the value closest but after the time of administration will be recorded. The following data will be recorded:

- Date/time of expiry of the study drug.
- Date/time of commencing the infusion of the study drug.
- Date/time of completion of infusion of the study drug.
- Study drug administration.

The following parameters will be recorded immediately prior to study drug administration and every 15 minutes for the duration of the infusion of study drug and every hour for the next 5 hours.

- FiO_2, PEEP, oxygen saturation and plateau pressure as well as arterial blood gas pH,PaO_2 and $PaCO_2$ if clinically available.
- Heart rate, systolic and diastolic blood pressure, vasopressor doses.

Temperature will be recorded directly prior to study drug administration and will be recorded every 15 minutes for the duration of the infusion of the study drug and every hour for the next 5 hours.

Day 1-14 (Daily Data)

Daily data for Day 1 should be collected after infusion of the IMP. FiO_2, PaO_2 and $PaCO_2$ will be recorded approximately every 12 hours on Days 2 and 3. All other daily measurements will be recorded and collected between 6-10am or as close to this time as possible, unless otherwise stated in the CRF. Daily data will be collected to day 14, or until ICU discharge, and will include but is not limited to:

- Determinants of the SOFA score
- Temperature
- Ventilation parameters including but not limited to: Mode of ventilation, minute volume, RR, mean airway pressure, plateau pressure, PEEP
- Arterial blood gas including but not limited to FiO_2, PaO_2, $PaCO_2$, pH, lactate
- Use of adjunctive therapies
- Renal replacement therapy
- Clinical laboratory assessments where collected as part of standard care: renal, liver function and CRP, haematological and coagulation parameters where possible.
- Overall fluid balance
- Adverse event assessment
- Echocardiography parameters (during phase 2 study) including but not limited to ventricular size and function and tricuspid annular plane systolic excursion (TAPSE) will be collected where possible (on day 4 only).

Day 4, 7 and 14

- Blood and urine will be taken on days 4, 7 and 14 (where possible)

Day 15 - 28

- Blood for anti-HLA antibodies will be collected on day 28 where possible

Day 90

- Adverse events will be collected up to day 90

Year 1

- Significant medical events
- Blood sample will be taken (where possible)

Year 2

- Significant medical events
- Blood sample will be taken (where possible)

The following data will also be collected:

- Date/time of extubation
- Date/time of re-intubation
- Date/time of discontinuation of mechanical ventilation (unassisted breathing)
- Date/time of critical care discharge
- Date/time of hospital discharge
- Date of death, including cause of death
- Final diagnosis on ICU discharge/death

Extubation is defined as first time being successfully free from an endotracheal tube or a tracheostomy tube for 48hrs.

Unassisted breathing i.e. no ventilatory support is defined as; extubated with supplemental oxygen or room air, or open T-tube breathing, or tracheostomy mask breathing, or CPAP without inspiratory pressure support for 48 hours. Patients receiving pressure support via non-invasive ventilation (except for sleep disordered breathing) or extra-corporeal lung support will be defined as receiving ventilatory support.

Discharge from critical care is defined as first discharge to a ward in the hospital or another hospital; a transfer between ICUs is not considered a discharge from critical care. Hospital discharge is the first date that the patient is discharged to home/community, a transfer between hospitals is not considered as a hospital discharge.

VFDs to day 28 are defined as the number of days from the time of initiating unassisted breathing to day 28 after study drug administration, assuming survival for at least 48 hours after initiating unassisted breathing and continued unassisted breathing to day 28. If a patient returns to assisted breathing and subsequently achieves unassisted breathing to day 28, VFDs will be counted from the end of the last period of assisted breathing to day 28. A period of assisted breathing lasting less than 24 hours and for the purpose of a surgical procedure will not count against the VFD calculation. If a patient was receiving assisted breathing at day 27 or dies prior to day 28, VFDs will be zero. Patients transferred to another hospital or other health care facility will be followed to day 28 to assess this endpoint.

Time to extubation will be counted from time of study drug administration to extubation.

Duration of ventilation will be counted from time of study drug administration to being successfully free from assisted breathing.

Duration of critical care and hospital stay will be counted from time of study drug administration to discharge.

12.3 Sampling Procedures for Exploratory Outcomes

Blood and Urine Sampling

Blood samples (where possible) will be collected as follows by trained study staff and processed according to sample processing guidelines: (if measurements for translational studies cannot be collected this will not be recorded as a protocol deviation)

Baseline – up to 40ml plus additional 20ml for monocyte or neutrophil isolation
Day 4 - up to 40ml plus additional 20ml for monocyte or neutrophil isolation
Day 7 – up to 25 ml
Day 14 – up to 25 ml
Day 28 – 5ml
Year 1 – 10ml
Year 2 – 10ml

Blood samples at year 1 and 2 will be collected where possible

Urine samples (where possible) will be collected as follows by trained study staff and processed according to sample processing guidelines:
Baseline – 10ml
Day 4 – 10ml
Day 7 – 10ml
Day 14 – 10ml

Samples will be managed according to the Sample Processing Guideline. In summary, samples will be labelled with the patient's unique Participant Study Number. After processing locally samples will be transferred to Queen's University Belfast (QUB). Samples will be stored at –70ºC until analysis. Samples will be stored beyond study completion in Queen's University Belfast. As new scientific data become available we will be able to use this resource of stored samples to investigate if this new data is relevant to ARDS pending additional ethical approval if required.

Translational studies

Measurements will include:

1. Systemic inflammatory responses will be assessed by the following:
   Plasma and serum inflammatory response biomarkers which may include but are not limited to measurement of plasma CRP, cytokines (including but not limited to TNFα, IL1β, IL6, IL8), lipocalins, proteases and antiproteases, adhesion and activation molecule expression (including but not limited to sICAM1), NETs, circulating miRNAs, extracellular vesicles, lipid mediators, RAGE ligands, and whole blood transcriptome will be undertaken. Specific cellular populations within the blood (using but not limited to cytospins and flow cytometry) and identification of transcriptome changes within these cell populations will be investigated.

2. Indices of pulmonary and systemic epithelial and endothelial function and injury will be assessed by the following:
   Plasma and serum biomarkers which may include but not be limited to measurement of RAGE, Ang I/II, SP-D, vWF, PCP3 as well as total protein, plasma albumin, α2-macroglobulin, and protein permeability (albumin:α2-macroglobulin ratio) will be undertaken. Urinary albumin/creatinine ratio and makers of extracellular matrix degradation including but not limited to desmosine will also be measured.

3. Coagulation
   Markers of coagulation which may include but not be limited to the following, will be measured: Thrombin-antithrombin (TAT), activated FVII-antithrombin complex, FXI-AT, FXII-AT, C3a, sC5b-9, tissue factor, protein C, thrombomodulin and plasminogen activator inhibitor1 and thromboelastogram (TEG). Platelets, d-dimer and fibrinogen will be measured.

4. Anti-HLA antibodies
   Two Luminex® assays will be undertaken; an initial antibody screen with Luminex® multi-antigen beads to detect class I and class II MHC antibodies followed by a Luminex® single antigen bead assay to determine the specificity of any antibody detected. The study samples will be analysed in an NHS clinical laboratory. These will be measured on day 0 samples and samples taken at day 28.

Samples from subjects may also be tested on primary cultures of fresh human neutrophils, monocytes and macrophages as well as mesenchymal stromal cells to provide mechanistic insights. Measurements may include but will not be limited to the measurement of cell activation (shape change, MSC surface marker expression, CD11b surface expression, superoxide release), adhesion and transmigration, cytokine release and MMP production, rate of apoptosis and their ability to phagocytose.

Monocytes or neutrophils will be isolated from blood at baseline. Cells will be stimulated (as monocytes) or matured for 5-7 days to produce monocyte-derived macrophages (MDMs). Cells (monocytes or MDMs) will be stimulated with LPS or other inflammatory stimuli to identify

mechanisms modulating inflammatory responses in these cells during ARDS. The effect of MSCs on cytokine production and their regulation will be measured by techniques including ELISA, multiplex, western blot, transcription factor assays and gene expression.

12.4 Follow Up Visits and Procedures

The CTU will collect mortality data at day 28 and day 90 (if discharged from hospital).

Participant Follow-Up

After discharge from hospital, participant survival and significant medical events at 1 and 2 years post study drug administration will be determined either by telephone interview, review of medical notes or the electronic health care record by site staff. In addition contact with the GP or NHS Digital (if available in that region), will be undertaken centrally by CTU staff.

Where possible patients will have follow-up blood samples taken at 1 and 2 years post study drug administration. Patients will be contacted by a member of the trial team and arrangements will be made for attendance at a suitable healthcare facility to have samples taken.

After being informed of a participant's discharge, the CTU will send a note thanking them for their participation in the study and reminding them we will be back in contact for follow-up. Study participants will be asked to let the CTU know if they move house at any time after hospital discharge; NHS Digital will enable us to locate patients who move without informing the CTU.

13 DATA COLLECTION AND MANAGEMENT

13.1 Data Collection

To ensure accurate, complete and reliable data are collected, the CTU will provide training to site staff in the format of investigator meetings and/or site initiation visits.

All data for an individual patient will be collected by the PI or designee and recorded in source documents/electronic CRF for the study. For routinely collected clinical data the NHS record will be the source document. Patient identification on the CRF will be through their unique participant study number, allocated at the time of enrolment. Data will be collected and recorded on the electronic CRF by the PI or designee as per the CRF submission guidelines.

If the participant is transferred to another hospital the PI or designated member of the site study team will liaise with the receiving hospital to ensure complete data capture as per CRF instruction. If this is not possible, the primary outcome must be collected as a minimum.

Data censorship for each trial participant will occur 90 days post study drug administration.

13.2 Data Management

Following the entry of patient data into the study database, the data will be processed as per the CTU Standard Operating Procedures (SOPs) and the study specific Data Management Plan (DMP). Data queries will be generated electronically for site staff to clarify data or provide missing information. The designated site staff will be required to respond to these queries. All queries will be responded to/resolved within the study database and amended in the study database.

13.3 Data Quality

The CTU will provide training to site staff on trial processes and procedures including CRF completion and data collection.

On-site monitoring visits during the trial will check the accuracy of entries on the electronic CRF against the source documents, the adherence to the protocol, procedures and Good Clinical Practice (GCP).

Quality control is implemented by the CTU in the form of Standard Operating Procedures (SOPs), which are defined to encompass aspects of the clinical data management process, and to ensure standardisation and adherence to International Conference of Harmonisation Good Clinical Practice (ICH-GCP) guidelines and regulatory requirements.

Data validation will be implemented and discrepancy reports will be generated following data entry to identify discrepancies such as out of range, inconsistencies or protocol deviations based on data validation checks programmed in the clinical trial database.

A Data Monitoring & Ethics Committee (DMEC) will be convened for the study to carry out reviews of the study data at staged intervals during the study.

14 STATISTICAL CONSIDERATIONS

14.1 Sample Size

The phase 1 trial will recruit up to 18 participants.

Although the primary focus of the phase 2 trial is safety, there is, however, power to detect a difference in physiological outcomes.

The sample size for the phase 2 REALIST trial was 60 patients with ARDS (30 in each of the ORBCEL-C and placebo groups). Although this can be regarded as an indicative sample size for the planned study of patients with ARDS due to COVID-19, the larger the number randomised, the more accurate and definitive the trial's results will be. However, the numbers that can be randomised will depend critically on how large the pandemic becomes and the simplicity of the trial processes. With this in mind, the recruitment, randomisation, treatment, outcome measurement and reporting, and follow-up processes will be as streamlined as possible.

The primary efficacy outcome measure will be the difference in oxygenation index (OI) between the ORBCEL-C and placebo treated groups at day 7. Based on our data from a recently completed clinical trial in ARDS, the mean (standard deviation; SD) OI at day 7 in patients with ARDS is 62(51)cmH_2O/kPa [4]. To allow 1:1 recruitment (ORBCEL-C vs placebo) a sample size of 56 subjects will have 80% power at a two-tailed significance level of 0.05 using a two-sample t-test to detect a clinically significant difference of 39 cmH_2O/kPa in OI between groups. In a previous phase 2 study of similar size, we have found that an intervention can demonstrate a change in OI of a similar magnitude confirming a treatment effect of this size can be achieved [4]. Although we anticipate few withdrawals or loss to follow-up we have allowed for this in the sample size calculation. In previous UK multicentre studies in the critically ill <3% withdrew consent or were lost to follow-up [4, 65] and on this basis a conservative drop-out rate of 5% has been estimated. Therefore a total of 60 evaluable patients who have received study drug (30 patients in the ORBCEL-C and 30 in the placebo group) will be recruited.

14.2 Analysis Population

In phase 2 the primary analysis will be conducted on outcome data obtained from randomised participants who receive at least some of their randomly allocated treatment. It is possible that some subjects may not receive the full treatment dose. Therefore a secondary analysis will be undertaken on the population who receive the complete treatment dose. An additional analysis will also be conducted on patients who have a confirmed PCR diagnosis of COVID-19. A priori defined subgroup analyses will be undertaken based on severity of inflammation as measured by plasma CRP and ferritin.

14.3 Statistical Methods

For the Phase 1 trial no formal statistical analysis will be performed on safety data. The primary analysis will be descriptive and will focus on adverse events. The number of pre specified cell infusion associated events will also be reported. Descriptive analysis of pulmonary and non-pulmonary organ function will also be undertaken. We plan to publish the data from the phase 1 study.

The maximal tolerated dose up to 400×10^6cells will be proposed by the TMG and approved by the DMEC prior to use in the Phase 2 randomised controlled clinical trial.

For the Phase 2 trial, adverse events and prespecified cell infusion associated events will be reported as for the Phase 1 study in a descriptive analysis. For continuously distributed outcomes, differences between groups will be tested using independent samples t-tests and analysis of covariance with transformations of variables to normality if appropriate, or non-parametric equivalents. Chi-square tests (or Fisher's Exact tests) will be used for categorical variables. A p value of 0.05 will be considered as significant.

Correlations between changes in the biological markers measured and physiological and clinical outcomes will be assessed by appropriate graphical and statistical methods including Pearson's (or Spearman's) correlation coefficient.

A final analysis and report of the Phase 1 study is planned following the last patient's 90 day follow up. A final analysis and report of the phase 2 study is planned following the last patient's 90 day follow up. The 2 year follow up data will be published thereafter and will be an important long term outcome. Detailed statistical analysis plans for phase 1 and phase 2 will be written and approved by the independent DMEC prior to any analysis.

All the power calculations and methodology for data analysis have been confirmed by the trial statistician from the Northern Ireland Clinical Trials Unit (NICTU).

14.4 Missing Data

Every effort will be made to minimise missing baseline and outcome data in this trial. The level and pattern of the missing data in the baseline variables and outcomes will be established by forming appropriate tables and the likely causes of any missing data will be investigated. This information will be used to determine whether the level and type of missing data has the potential to introduce bias into the analysis results for the proposed statistical methods, or substantially reduce the precision of estimates related to treatment effects. If necessary, these issues will be dealt with using multiple imputation or Bayesian methods for missing data as appropriate.

15 PHARMACOVIGILANCE

15.1 Definition of Adverse Events

The European Clinical Trials Directive 2001/20/EC and applicable clinical trial regulations set out the legal requirements for adverse event recording, management and reporting of clinical trials.

Table 3: Terms and Definitions for Adverse Events

Term	Definition
Adverse Event<br>(AE)	Any untoward medical occurrence in a participant to whom a medicinal product has been administered, including occurrences which are not necessarily caused by or related to that product.
Adverse Reaction<br>(AR)	Any untoward and unintended response in a participant to an investigational medicinal product, which is related to any dose administered to that participant.
Unexpected Adverse Reaction<br>(UAR)	An adverse reaction the nature and severity of which is not consistent with the information about the medicinal product in question set out in the Summary of Product Characteristics (SPC) or Investigator's Brochure (IB) for that product.
Serious Adverse Event (SAE)<br>Serious Adverse Reaction (SAR)<br>Suspected Unexpected Serious Adverse Reaction (SUSAR)	Respectively, any adverse event, adverse reaction or unexpected adverse reaction that:<br><br>• results in death<br>• is life-threatening<br>• requires hospitalisation or prolongation of existing hospitalisation*<br>• results in persistent or significant disability or incapacity<br>• consists of a congenital anomaly or birth defect<br><br>Any other 'Important medical event(s)' that carries a real, not hypothetical, risk of one of the outcomes above.

*Hospitalisation is defined as an inpatient admission regardless of length of stay, even if the hospitalisation is a precautionary measure for continued observation. Hospitalisations for a pre-existing condition, including elective procedures that have not worsened, do not constitute a SAE.

15.2 Assessment of Adverse Events

The PI or designee is responsible for recording AEs observed. The PI or designee must assess all AEs for seriousness, causality, severity and if the adverse event is related to the study drug for expectedness.

15.3 Assessment of Causality

The PI or designee should make an assessment of causality, i.e. the extent to which it is believed that the event may be related to the study drug.

Category	Definition
Definitely*	Temporal relationship of the onset, relative to administration of the product, is reasonable and there is no other cause to explain the event, or a re-challenge (if feasible) is positive.
Probably*	Temporal relationship of the onset of the event, relative to the administration of the product, is reasonable and the event is more likely explained by the product than any other cause.
Possibly*	Temporal relationship of the onset of the event, relative to administration of the product, is reasonable but the event could have been due to another, equally likely cause.
Unlikely	Temporal relationship of the onset of the event, relative to administration of the product, is likely to have another cause which can by itself explain the occurrence of the event.
Not Related	Temporal relationship of the onset of the event, relative to administration of the product, is not reasonable or another cause can by itself explain the occurrence of the event.

* Where an event is assessed as possibly, probably or definitely related, the event is an AR.

15.4 Assessment of Severity

The PI or designee should make an assessment of severity for each AE according to the following categories:

**Category**	**Definition**
Mild<br>(Grade 1)	A reaction that is easily tolerated by the trial participant, causing minimal discomfort and not interfering with every day activities.
Moderate<br>(Grade 2)	A reaction that is sufficiently discomforting to interfere with normal everyday activities.
Severe<br>(Grade 3)	A reaction that prevents normal everyday activities.
Life Threatening<br>(Grade 4)	A reaction that has life threatening consequences; urgent intervention indicated.
Death<br>(Grade 5)	A reaction that results in death.

15.5 Assessment of Seriousness

The PI or designee should make an assessment of seriousness i.e. is this is an adverse event, adverse reaction or suspected unexpected adverse reaction that:

- Resulted in death.
- Is life-threatening.
- Requires hospitalisation or prolongation of existing hospitalisation.
- Results in persistent or significant disability or incapacity.
- Consists of a congenital anomaly or birth defect.
- Is any other important medical event(s) that carries a real, not hypothetical, risk of one of the outcomes above.

15.6 Assessment of Expectedness

The PI or designee is required to make an assessment of expectedness if the event is possibly, probably or definitely related to the study drug. Although MSCs have previously been administered to patients with ARDS (47) as this specific MSC product has not been administered to patients with ARDS before, there are no expected events and all ARs will be considered unexpected. Therefore all SARs will be considered as SUSARs.

15.7 Adverse Event Reporting Period

The AE reporting period for the trial begins upon the signature of informed consent and ends 90 days following the last administration of the study drug.

All AEs and SAEs that occur during this time will be followed until they are resolved or are clearly determined to be due to a patient’s stable or chronic condition or intercurrent illness(es).

15.8 Adverse Event Reporting

As REALIST is recruiting in a population that is already in a life-threatening situation, it is expected that many of the patients will experience AEs. Events that are expected in this population (i.e. events in keeping with the underlying condition) should not be reported as AEs.

Examples of such adverse events include transient hypoxemia, agitation, delirium, organ failure, nosocomial infections, skin breakdown, and gastrointestinal bleeding. Such events will not be considered reportable adverse events unless the event is considered by the investigator to be associated with the study drug, or unexpectedly severe or frequent for an individual patient with ARDS.

The following pre-specified adverse events occurring within 6 hours of the start of infusion will be collected:

1. An increase in vasopressor dose greater than or equal to the following:
   a. Norepinephrine: 0.1 mcg/kg/min
   b. Epinephrine: 0.1 mcg/kg/min
   c. Commencement of any vasopressor including norepinephrine, epinephrine, vasopressin, phenylephrine, and dopamine
2. New ventricular tachycardia, ventricular fibrillation or asystole
3. New cardiac arrhythmia requiring cardioversion
4. Hypoxemia requiring an increase in FiO2 of 0.2 or more and an increase in PEEP of 5 or more to maintain Sp02 in the target range
5. Clinical scenario consistent with transfusion incompatibility or transfusion-related infection (e.g. urticaria, new bronchospasm).

The following pre-specified adverse events occurring within 24 hours of the start of infusion will be collected:

1. Any death.
2. Any cardiac arrest.
3. Temperatures recorded as >38.5ºC or temperatures that are recorded as >38.5ºC prior to study drug administration and have increased by ≥1ºC.

The investigator should attempt, if possible, to establish a diagnosis based on the subject's signs and symptoms. When a diagnosis for the reported signs or symptoms is known, the investigator should report the diagnosis as the AE, rather than reporting the individual symptoms.

The investigator should follow all AEs observed during the study until they are resolved or stabilised, or the events are otherwise explained.

All AEs should be treated appropriately. The action taken to treat the AE and the outcome will be recorded in the CRF.

All AEs, ARs and UARs should be reported on the AE Form within the CRF.

These events will be included as part of the safety analysis for the trial and do not require expedited reporting to the CTU.

Any SAE considered at least possibly related to the study drug will be reported by the investigators whenever they become aware of it. An SAE will be defined as related to the IMP (ie a SAR) if assessed as being possibly, probably or definitely related to the IMP.

All SAEs, SARs and SUSARs should be reported using the SAE Reporting Form and must be reported to the CTU within 24 hours of becoming aware of the event to

clinicaltrials@nictu.hscni.net. All SAEs, SARS and SUSARs should also be reported on the AE Form within the CRF.

The CTU is responsible for reporting SAEs to the Sponsor, REC and MHRA within the required timelines as per the regulatory requirements. A fatal or life threatening SUSAR must be reported within 7 days after the CTU has first knowledge of such an event. Relevant follow up information will be sought and communicated within an additional 8 days. All other SUSARs will be reported to MHRA and REC within 15 days after the knowledge of such an event.

Dose limiting toxicity in the phase 1 trial will be defined as any SAR.

Trial specific guidelines will provide details of the communication plan to provide the study team at each site with information on serious adverse events occurring during the course of the trial.

15.9 Recording and Reporting Urgent Safety Measures

The Sponsor and investigator may take appropriate urgent safety measures to protect clinical trial subjects from any immediate hazard to their health and safety. The investigator may implement urgent safety measures without prior approval from the REC or MHRA.

When a PI becomes aware of information that necessitates an urgent safety measure, they should phone the MHRA Clinical Trials helpline on 020 3080 6456 and discuss the issue with a safety scientist or medical assessor immediately after an urgent safety measure has been implemented.

The PI or designee should report the urgent safety measure to the CTU immediately, using the dedicated email address clinicaltrials@nictu.hscni.net.

The CTU will report the urgent safety measure to the Chief Investigator and to the Sponsor immediately, using the dedicated email address, clinical.trials@belfasttrust.hscni.net.

The CI will notify the MHRA and the REC providing full details of the information they have received and the decision making process leading to the implementation of the urgent safety measure within 3 days.

The PI or designee should respond to queries from the Sponsor or Chief Investigator immediately to ensure the adherence to reporting requirements to REC and MHRA.

15.10 Pregnancy Reporting

Pregnancy is not considered an AE or SAE, however an abnormal outcome would be. Therefore the PI or designee must collect pregnancy information for female participants, and for females who become pregnant while their partners are participating in the trial. Consent should be obtained to follow up the pregnancy from the female partners of male participants.

The pregnancy reporting period for the trial is from the commencement of the study drug until 28 days post study drug administration. The PI or designee should complete and submit the Pregnancy Reporting Form to the CTU by email (clinicaltrials@nictu.hscni.net) within 14 days of being made aware of the pregnancy.

Any pregnancy that occurs in a participant or participant’s partner during the reporting period should be followed to outcome. Follow up/outcome information should be provided to the CTU as soon as it becomes available.

16 DATA MONITORING

16.1 Data Access

The agreement with each PI will include permission for trial related monitoring, audits, ethics committee review and regulatory inspections, by providing direct access to source data and trial related documentation. Agreement/consent from patients/Personal Legal Representative/Professional Legal Representative as appropriate for this will also be obtained. The patient's confidentiality will be maintained and will not be made publicly available to the extent permitted by the applicable laws and regulations.

16.2 Monitoring Arrangement

The CTU will be responsible for trial monitoring. On-site monitoring visits will be conducted in accordance with the trial monitoring plan. The frequency and type of monitoring will be detailed in the monitoring plan and agreed by the trial Sponsor.

Before the trial starts at a participating site, an initiation "visit" will take place to ensure that site staff are fully aware of the trial protocol and procedures. Given the impact of the COVID-19 pandemic on, for example, travel and access to sites, these "visits" might take place remotely. Checks will take place to ensure all relevant essential documents and trial supplies are in place. Monitoring during the trial will check the accuracy of data entered into the CRF against source documents, adherence to the protocol, procedures and GCP, and the progress of patient recruitment and follow up.

The PI or designee should ensure that access to all trial related documents including source documents are available during monitoring visits. The extent of source data verification (SDV) will be documented in the monitoring plan.

17 REGULATIONS, ETHICS AND GOVERNANCE

The trial will comply with the principles of GCP, the requirements and standards set out in the Research Governance Framework and the Medicines for Human Use (Clinical Trials) Regulations 2004 and subsequent amendments, and any new relevant legislation or regulations introduced because of the COVID-19 pandemic.

17.1 Regulatory and Ethical Approvals

The trial will be conducted in accordance with the ethical principles that have their origin in the Declaration of Helsinki. The protocol will be approved by a Research Ethics Committee (REC). A clinical trial authorisation (CTA) will be obtained from the Medicines for Healthcare products Regulatory Agency (MHRA) before the start of the trial.

The trial protocol was prepared in compliance with the SPIRIT 2013 statement (69). The trial will be registered at www.clinicaltrials.gov and the European Union Drug Regulating Authorities Clinical Trials (EudraCT) database.

17.2 Ethical Considerations

The vulnerability of this study group is fully appreciated and every effort will be undertaken to protect their safety and well-being, in line with The Medicines For Human Use (Clinical Trials) Regulations 2004 and subsequent amendments and the Research Governance Framework.

17.3 **Protocol Compliance**

The investigators will conduct the study in compliance with the protocol given approval/favourable opinion by the REC and the MHRA.

A protocol deviation is defined as an incident which deviates from the normal expectation of a particular part of the trial process. Any deviations from the protocol will be fully documented on the protocol deviation form in the CRF.

A serious breach is defined as a deviation from the trial protocol or GCP which is likely to effect to a significant degree:

(a) the safety or physical or mental integrity of the subjects of the trial; or
(b) the scientific value of the trial

The PI or designee is responsible for ensuring that any potential serious breaches are reported directly to the CTU within one working day using the dedicated email address clinicaltrials@nictu.hscni.net. The CTU will notify the CI and Sponsor immediately to ensure the adherence to reporting requirements to REC and MHRA where a serious breach has occurred.

Protocol compliance will be monitored by the CTU who will undertake site visits to ensure that the trial protocol is adhered to and that necessary paperwork (e.g. CRF's, patient consent) is being completed appropriately.

17.4 **Protocol Amendments**

All protocol amendments will be undertaken in accordance with the regulatory requirements. Substantial changes to the protocol will require REC and MHRA approval prior to implementation, except when modification is needed to eliminate an immediate hazard(s) to patients.

17.5 **Good Clinical Practice**

The trial will be carried out in accordance with the principles of the International Conference on Harmonisation Good Clinical Practice (ICH-GCP) guidelines (www.ich.org). All members of the trial team will be required to have completed GCP training.

17.6 **Indemnity**

The BHSCT will provide indemnity for any negligent harm caused to patients through the Clinical Negligence Fund in Northern Ireland. QUB will provide indemnity for negligent and non-negligent harm caused to patients by the design of the research protocol.

17.7 **Patient Confidentiality**

In order to maintain confidentiality, all CRFs, questionnaires, study reports and communication regarding the study will identify the patients by their unique participant study number and initials only. Patient confidentiality will be maintained at every stage and will not be made publicly available to the extent permitted by the applicable laws and regulations.

17.8 Data Access

All essential documentation i.e. the Investigator Site file (ISF) and source data will be stored by sites. The TMF and associated trial data will be stored by the CTU in conformance with the applicable regulatory requirements and access to stored information will be restricted to authorised personnel. Following the publication of the primary and secondary study outcomes, there may be scope to conduct additional analyses on the data collected. In the event of publications arising from such analyses, those responsible will need to provide the CI with a copy of any intended manuscript for approval prior to submission.

17.9 Record Retention

The site PI will be provided with an ISF by the CTU and will maintain all trial records according to GCP and the applicable regulatory requirements. The PI is responsible for the archiving of essential documents at local sites in accordance with the requirements of the applicable regulatory requirements, Sponsor and local policies. The PI has a responsibility to allow Sponsor access to archived data and can be audited by the Sponsor on request. Following confirmation from the Sponsor the CTU will notify the PI when they are no longer required to maintain the files. If the PI withdraws from the responsibility of keeping the trial records, custody must be transferred to a person willing to accept responsibility and this must be documented in writing to the CTU and Sponsor.

The TMF will be held by the CTU within the BHSCT and the essential documents that make up the TMF will be listed in a SOP. On completion of the trial, the TMF and study data will be archived by the CTU according to the applicable regulatory requirements and as required by the BHSCT as Sponsor.

17.10 Competing Interests

The research costs including the cost of the intervention are funded by the Wellcome Trust and the Research and Development Division of the Public Health Agency, Northern Ireland. The CI and members of the TMG have no financial or non-financial competing interests and the members of the DMEC/TSC will be asked to confirm that they have no conflict of interest. In the event that a DMEC/TSC member reports a conflict of interest, advice will be sought from the Sponsor.

18 DISSEMINATION/PUBLICATIONS

18.1 Publication Policy

The trial will be reported in accordance with the Consolidated Standards of Reporting Trials (CONSORT) guidelines (www.consort-statement.org).

We plan to publish our trial protocol and statistical analysis plan to ensure transparency in our methodology. The phase 1 will be published on completion. The phase 2 trial will be published when data on primary outcome is available and in keeping with best practice for COVID-19 research. Long term data and mechanistic data will also be reported although may form the basis of separate publications.

The study findings will be presented at national and international meetings with abstracts on-line. Presentation at these meetings will ensure that results and any implications quickly reach all of the UK intensive care community. This will be facilitated by our investigator group, which includes individuals in executive positions in the UK Intensive Care Society. We will comply

with the Wellcome Trust open access policy and publish the findings of the trial in peer-reviewed open access (via Pubmed) journals. This will secure a searchable compendium of these publications and make the results readily accessible to the public, health care professionals and scientists. Any rapid dissemination methods available for COVID-19 trials will be used.

A lay person's summary of the principal findings of the results will be sent to all patients involved in the study at their request. The most significant results will be communicated to the public through press releases. An on-going update of the trial will also be provided on the CTU website.

18.2 Authorship Policy

Authorship will be determined according to the internationally agreed criteria for authorship (www.icmje.org).

18.3 Data Sharing Statement

Requests for data sharing will be reviewed on an individual basis by the CI.

The study will comply with the good practice principles for sharing individual participant data from publicly funded clinical trials [67] and data sharing will be undertaken in accordance with the required regulatory requirements and in the spirit of open research into COVID-19 .

19 REFERENCES

1. Rubenfeld GD, Caldwell E, Peabody E, Weaver J, Martin DP, Neff M, et al. Incidence and outcomes of acute lung injury. N Engl J Med. 2005;353(16):1685-93.
2. Bellani G, Laffey JG, Pham T, Fan E, Brochard L, Esteban A, et al. Epidemiology, Patterns of Care, and Mortality for Patients With Acute Respiratory Distress Syndrome in Intensive Care Units in 50 Countries. JAMA. 2016;315(8):788-800.
3. Villar J, Blanco J, Anon JM, Santos-Bouza A, Blanch L, Ambros A, et al. The ALIEN study: incidence and outcome of acute respiratory distress syndrome in the era of lung protective ventilation. Intensive Care Med. 2011;37(12):1932-41.
4. McAuley DF, Laffey JG, O'Kane CM, Perkins GD, Mullan B, Trinder TJ, et al. Simvastatin in the acute respiratory distress syndrome. N Engl J Med. 2014;371(18):1695-703.
5. Herridge MS, Cheung AM, Tansey CM, Matte-Martyn A, Diaz-Granados N, Al-Saidi F, et al. One-year outcomes in survivors of the acute respiratory distress syndrome. N Engl J Med. 2003;348(8):683-93.
6. Herridge MS, Tansey CM, Matte A, Tomlinson G, Diaz-Granados N, Cooper A, et al. Functional disability 5 years after acute respiratory distress syndrome. N Engl J Med. 2011;364(14):1293-304.
7. Hughes M, MacKirdy FN, Ross J, Norrie J, Grant IS, Scottish Intensive Care S. Acute respiratory distress syndrome: an audit of incidence and outcome in Scottish intensive care units. Anaesthesia. 2003;58(9):838-45.
8. Gates S, Perkins GD, Lamb SE, Kelly C, Thickett DR, Young JD, et al. Beta-Agonist Lung injury TrIal-2 (BALTI-2): a multicentre, randomised, double-blind, placebo-controlled trial and economic evaluation of intravenous infusion of salbutamol versus placebo in patients with acute respiratory distress syndrome. Health Technol Assess. 2013;17(38):v-vi, 1-87.
9. Ventilation with lower tidal volumes as compared with traditional tidal volumes for acute lung injury and the acute respiratory distress syndrome. The Acute Respiratory Distress Syndrome Network. N Engl J Med. 2000;342(18):1301-8.
10. Checkley W, Brower R, Korpak A, Thompson BT, Acute Respiratory Distress Syndrome Network I. Effects of a clinical trial on mechanical ventilation practices in patients with acute lung injury. Am J Respir Crit Care Med. 2008;177(11):1215-22.
11. Li G, Malinchoc M, Cartin-Ceba R, Venkata CV, Kor DJ, Peters SG, et al. Eight-year trend of acute respiratory distress syndrome: a population-based study in Olmsted County, Minnesota. Am J Respir Crit Care Med. 2011;183(1):59-66.
12. Angus DC, Clermont G, Linde-Zwirble WT, Musthafa AA, Dremsizov TT, Lidicker J, et al. Healthcare costs and long-term outcomes after acute respiratory distress syndrome: A phase III trial of inhaled nitric oxide. Crit Care Med. 2006;34(12):2883-90.
13. Taylor RW, Zimmerman JL, Dellinger RP, Straube RC, Criner GJ, Davis K, Jr., et al. Low-dose inhaled nitric oxide in patients with acute lung injury: a randomized controlled trial. JAMA. 2004;291(13):1603-9.
14. Bernard GR, Wheeler AP, Arons MM, Morris PE, Paz HL, Russell JA, et al. A trial of antioxidants N-acetylcysteine and procysteine in ARDS. The Antioxidant in ARDS Study Group. Chest. 1997;112(1):164-72.
15. Taut FJ, Rippin G, Schenk P, Findlay G, Wurst W, Hafner D, et al. A Search for subgroups of patients with ARDS who may benefit from surfactant replacement therapy: a pooled analysis of five studies with recombinant surfactant protein-C surfactant (Venticute). Chest. 2008;134(4):724-32.
16. Thompson BT. Glucocorticoids and acute lung injury. Crit Care Med. 2003;31(4 Suppl):S253-7.
17. Presneill JJ, Harris T, Stewart AG, Cade JF, Wilson JW. A randomized phase II trial of granulocyte-macrophage colony-stimulating factor therapy in severe sepsis with respiratory dysfunction. Am J Respir Crit Care Med. 2002;166(2):138-43.

18. Ferguson ND, Cook DJ, Guyatt GH, Mehta S, Hand L, Austin P, et al. High-frequency oscillation in early acute respiratory distress syndrome. N Engl J Med. 2013;368(9):795-805.
19. Young D, Lamb SE, Shah S, MacKenzie I, Tunnicliffe W, Lall R, et al. High-frequency oscillation for acute respiratory distress syndrome. N Engl J Med. 2013;368(9):806-13.
20. Organisation WH. Coronavirus disease 2019 COVID-19 Situation Report 46 2020 [https://www.who.int/docs/default-source/coronaviruse/situation-reports/20200306-sitrep-46-covid-19.pdf?sfvrsn=96b04adf_2].
21. Ruan Q, Yang K, Wang W, Jiang L, Song J. Clinical predictors of mortality due to COVID-19 based on an analysis of data of 150 patients from Wuhan, China. Intensive Care Med. 2020.
22. Zhou F, Yu T, Du R, Fan G, Liu Y, Liu Z, et al. Clinical course and risk factors for mortality of adult inpatients with COVID-19 in Wuhan, China: a retrospective cohort study. Lancet. 2020.
23. Friedenstein AJ, Chailakhjan RK, Lalykina KS. The development of fibroblast colonies in monolayer cultures of guinea-pig bone marrow and spleen cells. Cell Tissue Kinet. 1970;3(4):393-403.
24. Caplan AI. Mesenchymal stem cells. J Orthop Res. 1991;9(5):641-50.
25. Pittenger MF, Mackay AM, Beck SC, Jaiswal RK, Douglas R, Mosca JD, et al. Multilineage potential of adult human mesenchymal stem cells. Science. 1999;284(5411):143-7.
26. Erices A, Conget P, Minguell JJ. Mesenchymal progenitor cells in human umbilical cord blood. Br J Haematol. 2000;109(1):235-42.
27. Gronthos S, Mankani M, Brahim J, Robey PG, Shi S. Postnatal human dental pulp stem cells (DPSCs) in vitro and in vivo. Proc Natl Acad Sci U S A. 2000;97(25):13625-30.
28. Williams JT, Southerland SS, Souza J, Calcutt AF, Cartledge RG. Cells isolated from adult human skeletal muscle capable of differentiating into multiple mesodermal phenotypes. Am Surg. 1999;65(1):22-6.
29. Zuk PA, Zhu M, Ashjian P, De Ugarte DA, Huang JI, Mizuno H, et al. Human adipose tissue is a source of multipotent stem cells. Mol Biol Cell. 2002;13(12):4279-95.
30. Aguilar S, Nye E, Chan J, Loebinger M, Spencer-Dene B, Fisk N, et al. Murine but not human mesenchymal stem cells generate osteosarcoma-like lesions in the lung. Stem Cells. 2007;25(6):1586-94.
31. Lalu MM, McIntyre L, Pugliese C, Fergusson D, Winston BW, Marshall JC, et al. Safety of cell therapy with mesenchymal stromal cells (SafeCell): a systematic review and meta-analysis of clinical trials. PLoS One. 2012;7(10):e47559.
32. Fischbach MA, Bluestone JA, Lim WA. Cell-based therapeutics: the next pillar of medicine. Sci Transl Med. 2013;5(179):179ps7.
33. Devaney J, Horie S, Masterson C, Elliman S, Barry F, O'Brien T, et al. Human mesenchymal stromal cells decrease the severity of acute lung injury induced by E. coli in the rat. Thorax. 2015;70(7):625-35.
34. Jackson MV, Morrison TJ, Doherty DF, McAuley DF, Matthay MA, Kissenpfennig A, et al. Mitochondrial Transfer via Tunneling Nanotubes is an Important Mechanism by Which Mesenchymal Stem Cells Enhance Macrophage Phagocytosis in the In Vitro and In Vivo Models of ARDS. Stem Cells. 2016;34(8):2210-23.
35. Krasnodembskaya A, Samarani G, Song Y, Zhuo H, Su X, Lee JW, et al. Human mesenchymal stem cells reduce mortality and bacteremia in gram-negative sepsis in mice in part by enhancing the phagocytic activity of blood monocytes. Am J Physiol Lung Cell Mol Physiol. 2012;302(10):L1003-13.
36. Krasnodembskaya A, Song Y, Fang X, Gupta N, Serikov V, Lee JW, et al. Antibacterial effect of human mesenchymal stem cells is mediated in part from secretion of the antimicrobial peptide LL-37. Stem Cells. 2010;28(12):2229-38.
37. Bernardo ME, Zaffaroni N, Novara F, Cometa AM, Avanzini MA, Moretta A, et al. Human bone marrow derived mesenchymal stem cells do not undergo transformation after long-term in vitro culture and do not exhibit telomere maintenance mechanisms. Cancer Res. 2007;67(19):9142-9.

38. Aggarwal S, Pittenger MF. Human mesenchymal stem cells modulate allogeneic immune cell responses. Blood. 2005;105(4):1815-22.
39. Curley GF, Ansari B, Hayes M, Devaney J, Masterson C, Ryan A, et al. Effects of intratracheal mesenchymal stromal cell therapy during recovery and resolution after ventilator-induced lung injury. Anesthesiology. 2013;118(4):924-32.
40. Curley GF, Hayes M, Ansari B, Shaw G, Ryan A, Barry F, et al. Mesenchymal stem cells enhance recovery and repair following ventilator-induced lung injury in the rat. Thorax. 2012;67(6):496-501.
41. McAuley DF, Curley GF, Hamid UI, Laffey JG, Abbott J, McKenna DH, et al. Clinical grade allogeneic human mesenchymal stem cells restore alveolar fluid clearance in human lungs rejected for transplantation. Am J Physiol Lung Cell Mol Physiol. 2014;306(9):L809-15.
42. Rojas M, Cardenes N, Kocyildirim E, Tedrow JR, Caceres E, Deans R, et al. Human adult bone marrow-derived stem cells decrease severity of lipopolysaccharide-induced acute respiratory distress syndrome in sheep. Stem Cell Res Ther. 2014;5(2):42.
43. Asmussen S, Ito H, Traber DL, Lee JW, Cox RA, Hawkins HK, et al. Human mesenchymal stem cells reduce the severity of acute lung injury in a sheep model of bacterial pneumonia. Thorax. 2014;69(9):819-25.
44. Chan MCW, Kuok DIT, Leung CYH, Hui KPY, Valkenburg SA, Lau EHY, et al. Human mesenchymal stromal cells reduce influenza A H5N1-associated acute lung injury in vitro and in vivo. Proceedings of the National Academy of Sciences. 2016;113(13):3621.
45. Loy H, Kuok DIT, Hui KPY, Choi MHL, Yuen W, Nicholls JM, et al. Therapeutic Implications of Human Umbilical Cord Mesenchymal Stromal Cells in Attenuating Influenza A(H5N1) Virus-Associated Acute Lung Injury. J Infect Dis. 2019;219(2):186-96.
46. Zheng G, Huang L, Tong H, Shu Q, Hu Y, Ge M, et al. Treatment of acute respiratory distress syndrome with allogeneic adipose-derived mesenchymal stem cells: a randomized, placebo-controlled pilot study. Respir Res. 2014;15:39.
47. Wilson JG, Liu KD, Zhuo H, Caballero L, McMillan M, Fang X, et al. Mesenchymal stem (stromal) cells for treatment of ARDS: a phase 1 clinical trial. Lancet Respir Med. 2015;3(1):24-32.
48. L; Shi H; Hu Z; Zhang F; Gao J; Liu H; Li X; Zhao Y; Yin K; He X; Gao Z; Wang Y; Yang B, Jin R; Stambler I; Lim L; Su H; Moskalev A; Cano A; Chakrabarti S; Min K; Ellison-Hughes G; Caruso C; Jin K; Zhao R. . Transplantation of ACE2- Mesenchymal Stem Cells Improves the Outcome of Patients with COVID-19 Pneumonia. Aging and Disease. 2020;0:216-28.
49. Moodley Y, Sturm M, Shaw K, Shimbori C, Tan DB, Kolb M, et al. Human mesenchymal stem cells attenuate early damage in a ventilated pig model of acute lung injury. Stem Cell Res. 2016;17(1):25-31.
50. Packham DK, Fraser IR, Kerr PG, Segal KR. Allogeneic Mesenchymal Precursor Cells (MPC) in Diabetic Nephropathy: A Randomized, Placebo-controlled, Dose Escalation Study. EBioMedicine. 2016;12:263-9.
51. DePalma M, Amirdelfan K, Bae H, Coric D. Spine Intervention Society – 2016 24th Annual Scientific Meeting Research Abstracts. Pain Medicine. 2016;17(8):1577-97.
52. Moll G, Rasmusson-Duprez I, von Bahr L, Connolly-Andersen AM, Elgue G, Funke L, et al. Are therapeutic human mesenchymal stromal cells compatible with human blood? Stem Cells. 2012;30(7):1565-74.
53. Poncelet AJ, Vercruysse J, Saliez A, Gianello P. Although pig allogeneic mesenchymal stem cells are not immunogenic in vitro, intracardiac injection elicits an immune response in vivo. Transplantation. 2007;83(6):783-90.
54. Hare JM, Fishman JE, Gerstenblith G, DiFede Velazquez DL, Zambrano JP, Suncion VY, et al. Comparison of allogeneic vs autologous bone marrow-derived mesenchymal stem cells delivered by transendocardial injection in patients with ischemic cardiomyopathy: the POSEIDON randomized trial. JAMA. 2012;308(22):2369-79.

55. Kaipe H, Carlson LM, Erkers T, Nava S, Mollden P, Gustafsson B, et al. Immunogenicity of decidual stromal cells in an epidermolysis bullosa patient and in allogeneic hematopoietic stem cell transplantation patients. Stem Cells Dev. 2015;24(12):1471-82.
56. Griffin MD, Ryan AE, Alagesan S, Lohan P, Treacy O, Ritter T. Anti-donor immune responses elicited by allogeneic mesenchymal stem cells: what have we learned so far? Immunol Cell Biol. 2013;91(1):40-51.
57. Patil S, Chen X, O'Brien T. Topical application of CD362+ human mesenchymal stem cells (cyndacel-M) seeded in Excellagen scaffold augments wound healing and increases angiogenesis in a diabetic rabbit ulcer model. 17th European Congress of Endocrinology; Dublin, Ireland: Endocrine Abstracts; 2015.
58. Seeley E, McAuley DF, Eisner M, Miletin M, Matthay MA, Kallet RH. Predictors of mortality in acute lung injury during the era of lung protective ventilation. Thorax. 2008;63(11):994-8.
59. Fort P, Farmer C, Westerman J, Johannigman J, Beninati W, Dolan S, et al. High-frequency oscillatory ventilation for adult respiratory distress syndrome--a pilot study. Crit Care Med. 1997;25(6):937-47.
60. Levels of Critical Care for Adult Patients. Intensive Care Society, London2009.
61. Force ADT, Ranieri VM, Rubenfeld GD, Thompson BT, Ferguson ND, Caldwell E, et al. Acute respiratory distress syndrome: the Berlin Definition. JAMA. 2012;307(23):2526-33.
62. Core Standards for Intensive Care Units. Intensive Care Society, London2013.
63. Craig TR, Duffy MJ, Shyamsundar M, McDowell C, O'Kane CM, Elborn JS, et al. A randomized clinical trial of hydroxymethylglutaryl- coenzyme a reductase inhibition for acute lung injury (The HARP Study). Am J Respir Crit Care Med. 2011;183(5):620-6.
64. Papazian L, Forel JM, Gacouin A, Penot-Ragon C, Perrin G, Loundou A, et al. Neuromuscular blockers in early acute respiratory distress syndrome. N Engl J Med. 2010;363(12):1107-16.
65. Gao L, Zhang Y, Hu B, Liu J, Kong P, Lou S, et al. Phase II Multicenter, Randomized, Double-Blind Controlled Study of Efficacy and Safety of Umbilical Cord-Derived Mesenchymal Stromal Cells in the Prophylaxis of Chronic Graft-Versus-Host Disease After HLA-Haploidentical Stem-Cell Transplantation. J Clin Oncol. 2016;34(24):2843-50.
66. Hoffmann TC, Glasziou PP, Boutron I, Milne R, Perera R, Moher D, et al. Better reporting of interventions: template for intervention description and replication (TIDieR) checklist and guide. BMJ. 2014;348:g1687.
67. Wiedemann HP, Wheeler AP, Bernard GR, Thompson BT, Hayden D, deBoisblanc B, et al. Comparison of two fluid-management strategies in acute lung injury. N Engl J Med. 2006;354(24):2564-75.
68. Carless PA, Henry DA, Carson JL, Hebert PP, McClelland B, Ker K. Transfusion thresholds and other strategies for guiding allogeneic red blood cell transfusion. Cochrane Database Syst Rev. 2010(10):CD002042.
69. Chan AW, Tetzlaff JM, Altman DG, Laupacis A, Gotzsche PC, Krleza-Jeric K, et al. SPIRIT 2013 statement: defining standard protocol items for clinical trials. Ann Intern Med. 2013;158(3):200-7.

20 APPENDICES

Appendix 1	P/F Ratio Reference Table for Inclusion Criteria
Appendix 2	ARDSNet PEEP/FiO_2 Table
Appendix 3	Guidelines for Management During Study Drug Infusion

Appendix 1: P/F Ratio Reference Table for Inclusion Criteria

FiO2	Maximum PaO2 if P/F ratio ≤ 27kPa
0.50	10.0 kPa
0.55	11.0 kPa
0.60	12.0 kPa
0.65	13.0 kPa
0.70	14.0 kPa
0.75	15.0 kPa
0.80	16.0 kPa
0.85	17.0 kPa
0.90	18.0 kPa
0.95	19.0 kPa
1.00	20.0 kPa

Appendix 2: ARDSNet PEEP/FiO2 Table

**FiO2**	0.3	0.4	0.4	0.5	0.5	0.6	0.7	0.7
**PEEP**	5	5	8	8	10	10	10	12

**FiO2**	0.7	0.8	0.9	0.9	0.9	1.0
**PEEP**	14	14	14	16	18	18-24

Appendix 3: Guidelines for Management During Study Drug Infusion

Adverse Reactions	Signs and Symptoms	Management
Hypersensitivity<br><br>Hypersensitivity to allogeneic plasma proteins.	• dyspnea<br>• hypotension<br>• fever<br>• urticaria<br>• tachycardia<br>• hypoxemia	• Pause MSC infusion<br>• Check vital signs and O_2 saturation<br>• Hydrocortisone IV 100-200 mg<br>• If severe adrenaline IV 1:10,000 50-100 microg (0.5-1ml).<br>• Increase supplemental O_2 if O_2 saturation is <93% and/or dyspnea<br>• Resume MSC infusion after reasonable resolution of signs and symptoms if considered safe to do so.
Leukoagglutination<br><br>Because some leukocytes may be present in the MSC preparation (although MSC purity will typically be >95%), leukoagglutination symptoms may occur.	• cough<br>• "tickle in throat"<br>• dyspnea<br>• hypoxemia<br>• chest pressure	• Pause MSC infusion.<br>• Check vital signs and O_2 saturation<br>• Increase supplemental O_2 if O_2 saturation is <93% and/or dyspnea.<br>• Resume MSC infusion after reasonable resolution of signs and symptoms.
Reactions to Cryopreservatives (e.g. DMSO)<br><br>DMSO is added to all MSC products being cryopreserved to protect the MSC from hypothermal damage. The infusion of DMSO has side effects and consequences that study personnel need to be aware of	• metallic/garlic taste<br>• nausea/vomiting<br>• flushing of face<br>• intestinal cramps<br>• acute hypertension<br>• bradycardia (first, second, or third degree atrioventricular block on electrocardiogram)	• Pause MSC infusion.<br>• Check vital signs and O_2 saturation.<br>• Administer additional anti-emetics if appropriate.<br>• If bradycardia, perform an electrocardiogram. If associated with hypotension, stop infusion and administer atropine 0.5-1mg.<br>• If bradycardia is present and ECG shows third degree atrio-ventricular block, consider placement of a temporary pacemaker. Consult Cardiology.<br>• Resume MSC infusion after reasonable resolution of signs and symptoms if considered safe to do so.
